# Ionic Hydrogel‐Based Moisture Electric Generators for Underwater Electronics

**DOI:** 10.1002/advs.202408954

**Published:** 2024-09-29

**Authors:** Daozhi Shen, Fangzhou Li, Jian Zhao, Rui Wang, Bin Li, Zechao Han, Linglan Guo, Peicheng Han, Dongqi Yang, Hyun Ho Kim, Yanjie Su, Zhixiong Gong, Limin Zhu

**Affiliations:** ^1^ School of Mechanical Engineering Shanghai Jiao Tong University Shanghai 200240 China; ^2^ National Center for Translational Medicine Shanghai Jiao Tong University Shanghai 200240 China; ^3^ School of Materials Engineering Shanghai University of Engineering Science Shanghai 201620 China; ^4^ University of Michigan – Shanghai Jiao Tong University Joint Institute Shanghai Jiao Tong University Shanghai 200240 China; ^5^ Department of Energy Engineering Convergence School of Materials Science and Engineering Kumoh National Institute of Technology Gumi 39177 Republic of Korea; ^6^ Department of Micro/Nano Electronics School of Electronics Information and Electrical Engineering Shanghai Jiao Tong University Shanghai 200240 China; ^7^ State Key Laboratory of Ocean Engineering School of Ocean and Civil Engineering Shanghai Jiao Tong University Shanghai 200240 China; ^8^ State Key Laboratory of Mechanical System and Vibration Shanghai Jiao Tong University Shanghai 200240 China

**Keywords:** flexibility, moist‐electric film generator, nanogenerator, self‐powered, underwater application

## Abstract

Ubiquitous moisture is of particular interest for sustainable power generation and self‐powered electronics. However, current moisture electric generators (MEGs) can only harvest moisture energy in the air, which tremendously limits the energy harvesting efficiency and practical application scenarios. Herein, the operationality of MEG from air to underwater environment, through a sandwiched engineered‐hydrogel device with an additional waterproof breathable membrane layer allowing water vapor exchange while preventing liquid water penetration, is expanded. Underwater environment, the device can spontaneously deliver a voltage of 0.55 V and a current density of 130 µA cm^−2^ due to the efficient ion separation assisted by negative ions confinement in hydrogel networks. The output can be maintained even under harsh underwater environment with 10% salt concentration, 1 m s^−1^ disturbing flow, as well as >40 kPa hydraulic pressure. The engineered hydrogel used for MEG also exhibits excellent self‐healing ability, flexibility, and biocompatibility. As the first demonstration of practical applications in self‐powered underwater electronics, the MEG device is successfully powering a wireless emitter for remote communication in water. This new type of MEG offers an innovative route for harvesting moisture energy underwater and holds promise in the creation of a new range of innovative electronic devices for marine Internet‐of‐Things.

## Graphical abstract

The moisture electricity generator based on a sandwiched engineered ionic hydrogel with an additional waterproof breathable membrane layer for underwater applications is reported. The device can harvest moisture energy underwater environment and is successfully demonstrated to power wireless emitter for remote communication in water, which provides new insights for self‐powered electronics design for marine Internet‐of‐Things.

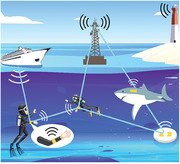

## 1 Introduction

Given the growing concern about the energy crisis and carbon emission, renewable off‐grid energy‐generation technologies such as photovoltaic,^[^ ^1^ ^]^ piezoelectric,^[^ ^2^ ^]^ and thermoelectric^[^ ^3^ ^]^ have been rapidly developed to meet the enormous demand for electric supply and the applications in powering electronics. Because moisture is ubiquitous in the natural environment as water covers two‐thirds of the Earth's surface,^[^ ^4^ ^]^ moisture electric generators (MEGs) that can directly harvest chemical energy from moisture have attracted significant attention in recent years.^[^ ^5^, ^6^, ^7^, ^8^ ^]^ Based on the charge flow induced by ions concentration gradient upon asymmetric water molecules absorbing,^[^ ^9^, ^10^ ^]^ MEGs exhibit green, renewable, and portable advantages.^[^ ^11^, ^12^ ^]^ These attributes render MEGs highly promising for reliable electricity generation and implementation in self‐powered devices.^[^ ^13^, ^14^, ^15^ ^]^

In this context, significant advancements have been witnessed in the development of MEGs using various materials such as graphene oxides,^[^ ^16^ ^]^ metal oxides,^[^ ^5^ ^]^ carbon nanoparticles,^[^ ^17^ ^]^ metal–organic frameworks,^[^ ^18^ ^]^ and polymers.^[^ ^19^ ^]^ With the various strategies including surface modification,^[^ ^20^ ^]^ chemical doping,^[^ ^21^ ^]^ multi‐layer structuring,^[^ ^22^ ^]^ the voltage output has been already realized from hundreds of millivolts of a single device to thousand volts level via large‐scale integration.^[^ ^23^ ^]^ The liquid‐solid contact charging effect^[^ ^24^, ^25^, ^26^ ^]^ has also been used for harvesting mechanical energy from water droplets. Due to its excellent hygroscopicity, flexibility, and mechanical properties, the hydrogel has been widely used for MEG devices^[^ ^27^ ^]^ and self‐powered sensors.^[^ ^28^, ^29^, ^30^ ^]^ So far, however, most MEGs are only capable of harvesting energy from moisture in the air,^[^ ^31^, ^32^ ^]^ a small amount of water source on Earth. The MEGs that can work underwater are lacking, making the vast energy from liquid water that accounts for the main proportion of water on Earth not harvestable through the current MEG technology. Additionally, the implementation of such generators with electronics to construct self‐powered systems for underwater applications is not achievable with existing MEGs. Meanwhile, most functional materials for electricity generation with moisture are not biocompatible,^[^ ^5^, ^33^ ^]^ which causes additional harm to the environment during applications.

**Figure 1:**
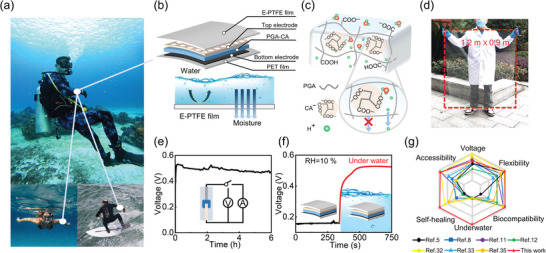
Concept and performance of the ionic‐confinement‐assisted hydrogel MEG with underwater operationality. a) Potential applications of self‐powered wearable MEG devices underwater for divers, swimmers, and surfers. Images are from the Pexels website, which provides free licensed images. b) Schematic details of the engineered‐hydrogel‐based MEG devices with E‐PTFE film for harvesting moisture underwater. c) The molecular structure and ionic confinement effect in PGA–CA hydrogel. d) Photograph of a large hydrogel film with a full size of 1.2 m × 0.9 m. e) The open‐circuit voltage of MEG sustained for over 6 h at RH of 90%. f) Voltage output of MEG when immersed in liquid water. g) Comparative summary of comprehensive performance with reported MEGs.

Herein, we report an efficient, self‐healable, biocompatible, flexible, and scalable MEG device that can directly harvest energy from moisture, but in an underwater environment, for the first time. The construction is based on a waterproof breathable membrane (WBM) containing micronized holes allowing water vapor to be transferred into an acid‐embedded hydrogel layer for electricity generation while preventing the liquid water penetration underwater. The moist‐active hydrogel is a polymeric network of polyglutamic acid (PGA) embedded with citric acid (CA) medium for confining large negative ions while facilitating small protons conduction. Due to this confinement‐assisted efficient ion separation, hydrophilic surface, and high moisture absorption, a single device can continuously deliver an open‐circuit voltage of 0.55 V and a peak current density of 130 µA cm^−2^ in water. The output can be maintained even under harsh underwater environment with 10% salt concentration, 1 m s^−1^ disturbing flow, and >40 kPa hydraulic pressure. Significantly, a novel underwater wireless communication system is demonstrated, which is powered by our complementary energy harvester. This work successfully expands harvestable moisture energy using MEGs from air to underwater environment (**Figure** **1**a), facilitating the creation of a wide range of self‐powered underwater electronics in potential applications such as emergency alarms, water environment monitoring, and underwater Internet‐of‐Things (IoTs).

## 2 Results and Discussion

A flexible moisture electric generator based on homogeneous moist‐active hydrogel with a waterproof breathable membrane (WBM) is designed for harvesting underwater moisture energy (shown in Figure 1b). The hydrogel consists of uniformly distributed polyglutamic acid (PGA) and citric acid (CA) medium, with CA embedded within the skeletons of PGA chains (Figure 1c). Such a unique design induces efficient electricity generation under a moist environment due to the following principles: 1) The hydrophilic polymer network enables excellent water molecule absorption from moisture. 2) While PGA already has abundant carboxyl (─COOH) groups for free H^+^ ions dissociating, the addition of CA dissociates more H^+^ ions due to enriched ─COOH groups in CA when interacting with water molecules. 3) Most importantly, the anions (─COO^−^) with relatively large sizes dissociated from CA are strongly confined in the 3D PGA networks, while the small H^+^ ions can move freely (Figure 1c). We emphasize that this ionic confinement is the key to efficient electricity generation. Without the 3D network confinement, the anions from CA can also move along with H^+^ diffusion, which cancels the pure electric field across the film and reduces electricity output, as we shall discuss in the context later. Similarly, the output will also be compressed when there is no additive CA for enormous H^+^ donation. The mixing of PGA and CA was dropped onto an inert electrode film, then dried under 25 °C and 30% relative humidity (RH) for 12 h to obtain a 40 µm thick hydrogel film (Figure S1, Supporting Information). A free‐standing, flexible, and transparent hydrogel film with a size of 1.2 m × 0.9 m can be obtained (Figure 1d), which can be tailored and assembled for large‐scale and low‐cost fabrication.

The PGA is a chain of amino acids linked together and can be derived from natural products such as fermented soybeans.^[^ ^34^ ^]^ Thus, this hydrogel material has excellent biocompatibility and does no harm to the natural environment. The absorption peaks of PGA–CA hydrogel at 1000, 1750, and 3250 cm^−1^ from Fourier transform infrared (FTIR) spectroscopy results (Figure S2, Supporting Information) represent the C─O, C═O, and ─OH bonds of ─COOH, respectively. Due to more ─COOH in CA, the intensity of these peaks in PGA: CA (5:1) is much stronger than that of PGA: CA (20:1). The abundant carboxyl groups in the amino acid chains give rise to the hydrophilic nature of PGA having a low contact angle of 40° as shown in Figure S3 (Supporting Information). The bonds were also characterized by the X‐ray photoelectron spectroscopy (XPS). From the C1s band of the film, the characteristic peaks at 284.8, 286, 287, and 289 eV correspond to C─C, C─O, C─N, and C═O, respectively. As shown in Figure S4 (Supporting Information), a peak of C─O is much more pronounced as compared to other peaks, especially to C─C peak, which results from the largely enhanced carboxyl groups due to the additive CA. As the carboxyl groups are the main reason for their hydrophilic nature, the film shows enhanced hydrophilic characteristics. Thus, the obtained contact angle can be as low as 25° at the proper mixing ratio of PGA and CA (Figure S3, Supporting Information). Such hydrophilic hydrogel can spontaneously adsorb water molecules from moisture effectively, facilitating the permeation and diffusion of water molecules.

A top electrode with meshed holes was attached to hydrogel with edges sealed (Figure S1, Supporting Information), ensuring that moisture ingress is confined solely from the upper surface. As the rich hydrophilic groups exist, the hydrogel can spontaneously adsorb water molecules from moisture and dissociate positively charged H^+^ ions.^[^ ^35^ ^]^ Due to the strong ionic confinement, an efficient ion concentration difference is formed from the asymmetry of moisture absorption, resulting in electric potential and current flow.^[^ ^26^, ^33^ ^]^ With the film sandwiched with electrodes that are directly exposed to moist air, the bare device structure can generate a voltage of 0.55 V at a RH of 90% (Figure 1e). As a big breakthrough, we further expand the operationality of MEG from air to underwater environment, a key innovation of this work. As expected, without the additional layer to protect the device, however, the output vanishes immediately when the bare device is immersed in water directly (Figure S5, Supporting Information), resulting in the immediate failure of output. Here, we innovatively solve this challenge by covering the MEG device with expanded polytetrafluoroethylene (E‐PTFE) as the waterproof breathable membrane (Figure S6, Supporting Information). This membrane has a layer‐stacked structure containing abundant small holes, allowing water vapor to be transferred into the hydrogel layer for electricity generation (Figure S6, Supporting Information). At the same time, the E‐PTFE can prevent liquid penetration from outside (Figure S7, Supporting Information). As a result, the designed MEG device can generate a stable output of up to 0.55 V when immersed in water (Figure 1f; Movie S1, Supporting Information), demonstrating its excellent operationality underwater environment.

Moreover, the hydrogel for MEG exhibits excellent self‐healing ability and biocompatibility. The hydrogel was scratched with a crack and then placed in a moist environment (RH = 90%). Optical microscopy images show that the crack region of broken hydrogel is well healed and self‐repaired in 60 min under a moist environment (Figure S8, Supporting Information). The detailed self‐healing process can be seen from the real‐time optical microscopy characterization (Movie S2, Supporting Information). The absorbed water drives the reconstruction of the damaged hydrogel by bridging the crack edges with the bonds link between functional groups.^[^ ^32^, ^36^ ^]^ In addition, as both PAG and CA is a non‐toxic, eco‐friendly, and non‐immunogenic biopolymers, the hydrogel is biocompatible and gives no harm to the natural environment. The comparison of comprehensive performances including underwater operability, biocompatibility, self‐healing ability, voltage output, flexibility, and materials accessibility of the reported MEGs is summarized in Figure 1g and Table S1 (Supporting Information).

**Figure 2:**
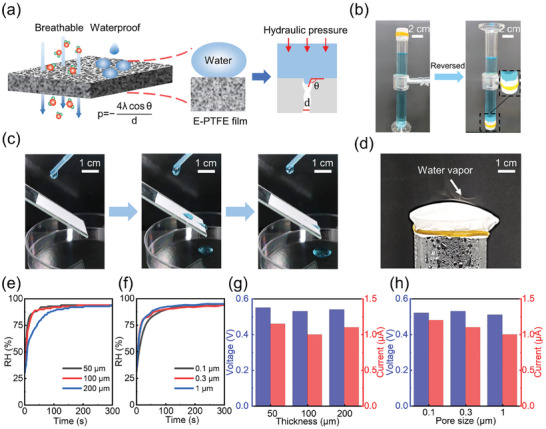
Properties of waterproof breathable membrane for MEGs working underwater. a) Working principles of E‐PTFE waterproof breathable membrane underwater. b) No water leakage from an E‐PTFE‐sealed glass cylinder. c) Optical image of a water drop impacting on porous E‐PTFE film, showing the hydrophobic surface. d) The capability of water vapor penetration through a P‐TFEP film. The change of RH in a chamber sealed with E‐PTFE film with different e) thicknesses and f) pore sizes when immersed in liquid water. The electricity output of MEG devices with different g) thicknesses and h) pore sizes of E‐PTFE film underwater.

The ability of MEGs to harvest moisture energy underwater is possible due to the design of a porous E‐PTFE waterproof breathable membrane that can effectively prevent liquid water penetration but allow water vapor transfer, as illustrated in **Figure** **2**a,b. According to Laplace's law,^[^ ^37^, ^38^ ^]^ the ability of porous film to prevent liquid water can be elucidated as:^[^ ^37^ ^]^

(1) $$P\;=\;-\frac{4\lambda \cos\theta }{d}$$

where *P* is the pressure required to pass through the membrane, *λ* is the surface tension of the liquid, *θ* is the water contact angle, and *d* is the pore diameter. Obviously, a membrane with a large contact angle and small pore size has a higher operational pressure underwater. Here, the E‐PTFE film has a highly hydrophobic surface (Figure 2c; Movie S3, Supporting Information), with a large contact angle (≈150°) (Figure S9, Supporting Information) as well as a small pore size (≤1 µm). Thus, the film can behave as an excellent barrier layer from liquid water penetration (Figure 2b; Figures S7, S10, and Movie S4, Supporting Information). Based on the equation, the calculated operational hydraulic pressure is around hundreds of kPa, corresponding to the depth of tens of meters underwater, which is also consistent with the measured values (Figure S11, Supporting Information). The surface is highly hydrophobic (Figure S12, Supporting Information), and a large water contact angle ≈150° can be maintained when the pore size is changed from 1 to 0.1 µm (Figure S9, Supporting Information). As a result, there is no liquid water leakage when placed into water for a long time. It should be emphasized that operational hydraulic pressure can be further improved to >5000 kPa (corresponding to 500 m underwater) theoretically, by optimizing the parameters of a waterproof breathable membrane.^[^ ^39^ ^]^

At the same time, as there are abundant small pores, the water vapor or moisture can be easily transferred through the membrane (Figure 2d), even if the pore size is down to 0.1 µm (Figure S13, Supporting Information). Thus, the humidity level can be increased rapidly (Figure 2e,f). This dynamic process is affected by the thickness of the E‐PTFE film. As shown in Figure 2e, the humidity level reaches ≈ 85% in 20 s and is then maintained when ≤ 100 µm. However, even though the final RH reached remains the same, the response speed is slightly slower when the film is thick (200 µm) (Figure S14, Supporting Information). This is due to the longer water molecules migration distance for thick film. Nevertheless, the water vapor can efficiently pass through E‐PTFE films to wet MEGs under different film thicknesses, pore diameters, and water hydraulic pressures (Figure 2e,f; Figure S15, Supporting Information) to activate efficient electricity generation. Thus, the output electricity can be stably maintained regardless of thicknesses (Figure 2g) and pore diameter (Figure 2h). To explore the effective life of the E‐PTFE membrane, we placed the E‐PTFE membrane in water for a long period of time. Since the membrane is relatively stable, there is no significant change in the electrical output of the MEG device after the long‐term exposure of E‐PFTE in water (Figure S16, Supporting Information). Impressively, the electricity output can be exceptionally maintained as long as >72 h under a wet environment as shown in Figure S17 (Supporting Information).

**Figure 3:**
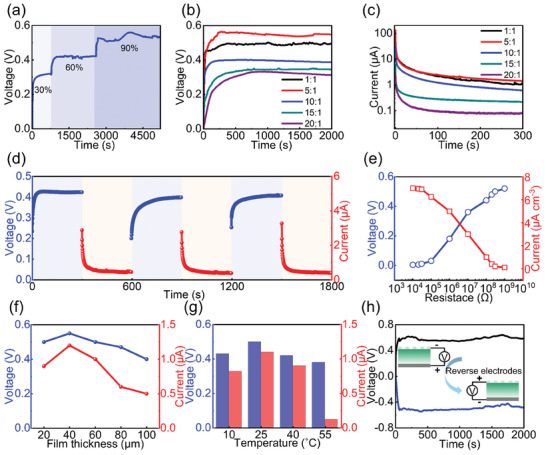
Electrical performance of the MEG. a) Voltage output of MEGs at different RHs at room temperature. b) Voltage and c) current output of MEGs at RH of 90% with different ratios of PGA to CA. d) Variation in voltage (blue) and current (red) of the MEG device at RH of 60%. e) Electrical output of MEG devices with external resistance loading ranging from 10^4^ to 10^9^ Ω. f) Output of MEGs with different hydrogel thickness at RH of 90% under room temperature. g) Output of MEGs at different temperatures with a hydrogel film thickness of 40 µm at 90% humidity. h) Output polarity test of MEGs.

To evaluate the response of the MEG to water vapor, the devices were placed in a chamber with controlled RH to mimic the wet conditions (Figure S18, Supporting Information). To eliminate the possible static electricity, all devices were connected in short circuits before the test. As shown in **Figure** **3**a, the open‐circuit voltage climbs gradually from zero and is stabilized at ≈0.33 V when the device is exposed to moisture with a low RH of 30%, exhibiting excellent output capability even in a relatively dry environment. Upon increasing the RH to 60%, the voltage increases gradually and is stabilized at 0.4 V. By further changing the RH to 90%, the device can yield an output as high as ≈0.6 V (Figure 3a). As shown in Figure S19 (Supporting Information), the electric performance shows high humidity dependence and the steady‐state output follows well with the humidity level. Furthermore, the output voltage decreases to a very low level when the RH is decreased to 10%, while it can be recovered to a high level when the RH is increased to 80% again (Figure S20, Supporting Information), showing robust output under an interval moist environment. More importantly, the MEGs have similar electricity output when different inert electrodes are used (Figure S21, Supporting Information), demonstrating that the electrochemical process should not be the dominant origination for electricity generation.

As discussed, the compositions of the polymeric network of PGA–CA can largely affect the electric performance of MEGs. The open‐circuit voltage is only 0.3 V when the additive CA concentration is low (with the PGA to CA ratio of 20:1) as shown in Figure 3b. As PGA chains have relatively lower ─COOH concentration compared to CA, this PGA‐dominated film has a limited number of hydrophilic groups for water absorption and proton donation, which gives low electricity output. Impressively, the output voltage increases with the further increase of CA, reaching almost 0.55 V at the PGA to CA ratio of 5:1. This mixing ratio also gives PGA‐CA film the lowest contact angle (Figure S3, Supporting Information). There are two reasons for the output enhancement: 1) the increased hydrophilic groups in CA promote water absorption, facilitating the interaction with hydrogel; 2) CA is confined within PGA chains, thus once the hydrogel is contacted with water molecules, the protons dissociate from CA while skeletons of CA still adhere to the PGA chains, efficiently forming an internal electric filed to improve the voltage output. However, the voltage output is slightly decreased once the CA is excessive, with the output of 0.5 V under the hydrogel mixing ratio of 1:1 (Figure 3b). This should be attributed to the movement of CA molecules along with the water and proton diffusion due to their weak adhesion to PGA chains at this low PGA concentration. Figure 3c summarizes the short‐circuit current with different PGA‐CA compositions. The device has a current output as high as ≈130 µA initially (red plot), and decays gradually to a steady state. This is due to the discharge of the accumulated charges. The device has maximized output at the mixing ratio of 5:1. Thus, we use this optimized composition for the following device characterization.

The self‐charging capability is essential for practical self‐powered applications when MEGs are used as a power source. The cyclic charging and discharging characteristics can be seen in Figure 3d. The MEG can deliver a stable voltage output of 0.4 V at RH of 60% over a long period of time. After the discharging with short‐circuit for 5 min, a drop in voltage is observed. Interestingly, the MEG can recharge itself and the voltage can be restored to its original voltage in 5 min, which is significantly faster than that of the state‐of‐the‐art MEG devices.^[^ ^8^, ^35^, ^40^ ^]^ This confirms the reliable self‐charging capability of the hydrogel‐based MEG, which helps to power external loads stably. The electrical output ability is also investigated by connecting the MEG device to different external resistive loads. As shown in Figure 3e, with the increase in loading resistance, the output voltage is gradually increased while the steady‐state current decreases.

The effect of PGA‐CA hydrogel thickness and ambient temperature on the performance of MEGs was also investigated. Both the output voltage and the current exhibit a nonlinear dependence on the film thickness. They both initially increase with the thickness of the hydrogel until the thickness reaches ≈40 µm. The output subsequently decreases with a further increase in thickness (Figure 3f). The observation of a peak in electric output demonstrates an optimized distance for the ion migration. When the film is too thin (<40 µm), the ion concentration gradient is hard to establish so the output is low. When it is too thick, the diffusion pathway for water molecules and protons is too long, leading to weak ion migration and decreased electric output. We also investigate the output of the device with different areas. As expected, the voltage remains constant with different sizes of MEG, while the output current scales linearly with the MEG area (Figure S22, Supporting Information). The output also has a temperature‐dependent response, with an operational temperature range of 10–55 °C (Figure 3g). This range covers the normal temperature range of the liquid water on Earth.^[^ ^41^ ^]^ As illustrated in Figure 3h, the lower moisture side of the MEG device always has a higher potential. By reversing the connection, the inverse output voltage is obtained, showing that the ion migration direction and electric output are directly related to the moisturizing direction.

**Figure 4:**
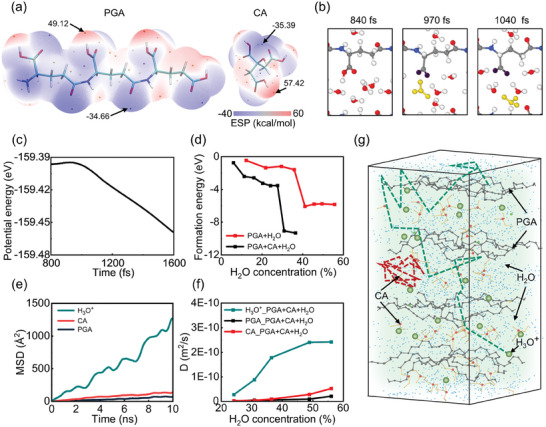
DFT and MD simulations. a) The electrostatic potential (ESP) distribution of CA and PGA by DFT calculations. Surface local minima and maxima of ESP are represented as orange and violet spheres, respectively. b) The snapshots of the PGA‐water system by AIMD simulation. Red, gray, white, and blue balls represent oxygen, carbon, hydrogen, and nitrogen, respectively. Yellow and purple balls are highlighted H_3_O^+^ and the oxygen of ─COO^−^ groups, respectively. c) The potential energy evolution during the AIMD simulation for the process in (b). d) The formation energy of the system versus water concentration by DFT calculations. e) The MSD of CA, PGA, and H_3_O^+^ in PGA‐CA hydrogel with water from classical MD simulation. The water concentration of the system is 39%. f) The diffusion coefficient of CA, PGA, and H_3_O^+^ versus water concentration. g) The snapshot of the system of PGA‐CA hydrogel with water. The water concentration is 39%. Green and orange balls are the mass centers of H_3_O^+^ and CA. The dashed lines with corresponding colors are the trajectories of H_3_O^+^ and CA during the 10 ns classical MD simulation, respectively.

To further explore the interaction between hydrogel and moisture, density functional theory (DFT) calculations and molecular dynamics (MD) simulations are conducted. The water affinities with PGA and CA are investigated by the molecular electrostatic potential (ESP) distributions on the molecular van der Waals (vdW) surface calculated by DFT, as illustrated in **Figure** **4**a. Both PGA and CA show high ESP charge distribution near the proton of ─COOH groups. The maximum and minimum ESP values of the PGA monomer unit are 61.92 and −41.19 kcal mol^−1^, respectively, while CA shows higher absolute ESP values of 57.42 and −35.39 kcal mol^−1^ (Figures S23 and S24, Supporting Information). With larger absolute values and more specific ─COOH groups (each CA molecule has 3 groups), the additive CA improves the moisture absorption capability of the mixed hydrogel. The interaction between the ─COOH groups and water molecules is captured by ab initio molecular dynamics (AIMD) simulation, as shown in the snapshots of Figure 4b. Initially, the water molecules are attracted by highly polarized ─COOH groups (left panel in Figure 4b). With an appropriate water concentration, free protons are then dissonated from ─COOH groups and can be bonded with waters nearby to form hydronium ions (H_3_O^+^), leaving negatively charged ─COO^−^ groups (middle panel in Figure 4b). Then the diffusion of the proton can be observed, by hopping through the network of water molecules via the Grotthuss Mechanism (right panel in Figure 4b).^[^ ^42^ ^]^ The entire dynamic process can be seen in Movie S5 (Supporting Information). As illustrated in Figure 4c, the potential energy of the system decreases gradually in this process, indicating that the above water‐PGA interaction is a naturally proton‐dissociation‐favored process. We also investigate the influence of water concentrations on the generation and diffusion of H_3_O^+^. The formation energy of the water‐PGA system decreases dramatically over 40%, meaning a proton‐dissociation‐favored reaction of water with PGA (red plot in Figure 4d; Figure S25, Supporting Information). Moreover, it is well known that, the density of the mixed system decreases gradually with the increase of water concentration till a dilute solution (Figure S26, Supporting Information). In the low concentration range (<55%), the protons diffusion coefficient increases gradually with water concentration (Figure S27, Supporting Information). This indicates that a higher RH boosts the generation and directional moving of hydronium ions and hence provides higher electricity, which is consistent with the output characteristics observed in Figure 3a.

The effect of additive CA on the dynamic water‐hydrogel interaction for the output performance of MEG is illustrated in Figure 4d–g. The formation energy of water molecules decreases more sharply even at a much smaller water concentration (Figure 4d; Figure S28, Supporting Information), indicating a more favorable water‐hydrogel interaction to release protons due to the addition of CA. We also quantitatively analyze the dynamic behavior of H_3_O^+^, PGA, and CA by extracting their mean square displacement (MSD) (Figure 4e; Figures S29 and S30, Supporting Information). As shown in Figures S29 and S30 (Supporting Information), the addition of CA largely increases the mobility of H_3_O^+^ in hydrogel with the water concentration less than 55%. Meanwhile, the mobility of H_3_O^+^ is significantly higher than that of PGA and CA (Figure 4e). In the system with a water concentration of 39%, the MSD curves of PGA and CA reach plateaus after 8.5 ns, with values stabilizing at 71.65 and 127.54 Å^2^, respectively. However, the MSD of H_3_O^+^ increases over 1200 Å^2^ in 10 ns, with H_3_O^+^ entering into the diffusive regime. As such, the diffusion coefficient of H_3_O^+^ is significantly larger than that of PGA and CA as shown in Figure 4f. These results demonstrate that both PGA and CA are confined to the original positions, while protons exhibit the capability of highly sustained diffusion in the hydrogel system.

**Figure 5:**
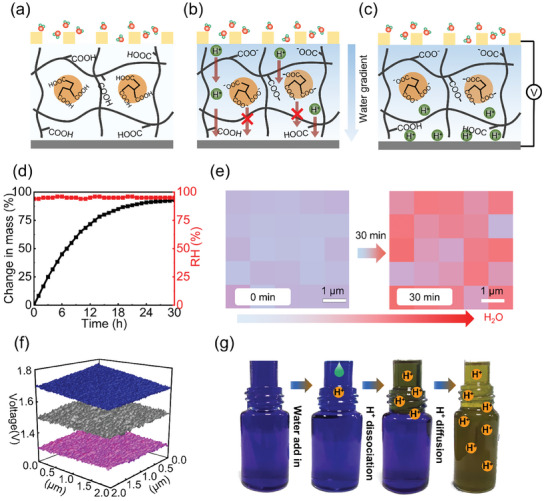
The working mechanism of the ionic‐confinement‐assisted hydrogel MEGs. a) PGA‐CA hydrogel with moisture. b) Negative charges confinement in the hydrogel networks. c) Electricity generation due to the positive protons diffusion and negative ions confinement. d) The change of hydrogel mass with time in the moist environment. e) Water absorption in hydrogel film characterized by Raman mapping spectroscopy. f) Kelvin Probe Force Microscopy (KPFM) characteristics of a hydrogel film under moisture. KPFM bias is set at 1.5 V, with one side absorbing moisture and the other side covered to remain dry. The potential of the wetting and non‐wetting surface is plotted in blue and pink, respectively. The gray mapping is the pristine film before the moisture was introduced. g) Dynamic changes in the ion diffusion process are visualized for moisture‐induced processes by dropping water. A hydrogel film is clamped between the open ends of two cylindrical containers containing bromothymol blue/isopropanol solutions.

The motions of protons and polymeric molecules can be clearly seen from the traces marked in Figure 4g and the dynamic process can be seen from Movie S6 (Supporting Information). Obviously, the protons can move more diffusively through the PGA chains due to their small size (see dashed green line). Due to the confinement of PGA chains on large molecules, however, the motion of large ─COO^−^ anions from CA is strongly localized (see dashed red line). In addition, the anions from PGA are also confined onto the unmovable chains. As a result, the freely directional motion of positively charged protons, together with the strong confinement of negatively charged ─COO^−^ anions promote the internal electric field across the hydrogel, producing an efficient electricity output. This mechanism is illustrated in **Figure** **5**a–c in detail. Clearly, when the excessive CA is added, the confinement effect to CA becomes compressed due to the low concentration of PGA chains and the mixed system gives lower output. This is consistent with the results in Figure 3b that there exists an optimal concentration of additive CA for efficient electricity output.

The above mechanism is also varied by the experimental results. We measured the mass of a hydrogel film under RH of 90%. Figure 5d shows that the film is highly adsorbable and the weight increases quickly with the water absorption from moisture. A doubled weight can be obtained after saturation in 30 h. Raman spectroscopy was carried out to analyze the surface chemical state. In a moist environment, there are two characteristic peaks of hydrogel located at 3300 and 2950 cm^−1^ (Figure S31, Supporting Information), corresponding to the H─O bonds from the absorbed water and the C─H bonds of intrinsic hydrogel, respectively. Thus, the ratio of these two peaks amplitude represents the intensity of water absorption.^[^ ^35^ ^]^ In situ spatially resolved Raman spectroscopy mapping was employed to track the absorption of water molecules on the hydrogel surface by taking the intensity ratio of the O─H bond to the C─H bond (Figure 5e). The color change clearly shows that the top surface of the hydrogel has captured abundant water molecules after being stored in a moist environment for 30 min. This result is also consistent with the small contact angle of hydrogel, showing the efficient water absorption capability due to the hydrophilic ─COOH groups. Thus, the MEG device can generate a high voltage due to the efficient water absorption from the moisture environment. On the contrary, there is no electric output when there are no holes in the top electrode for water vapor penetration (Figure S32, Supporting Information), indicating that humidity plays a key role in MEG power generation.

A Kelvin probe force microscope (KPFM) measurement for surface potential characterization was then carried out to validate the above‐proposed mechanism.^[^ ^35^ ^]^ To characterize the surface potential of the hydrogel membrane, we set the base voltage of the Kelvin probe force microscope to 1.5 V. In a dry environment, the surface potential of the hydrogel was almost stable at 1.5 V (Figure 5f), which means that there was no net surface charge in the original hydrogel. When moisture is introduced into the hydrogel, the negative surface potential of the moisture‐wetted side is +1.25 V. In contrast, the unwetted surface shows a positive potential of +1.7 V, which is due to the accumulation of positive charges. Clearly, the protons diffusion induced by the water molecules gradient uptakes the potential of the nonwetting surface but declines the potential of the wetting surface. Additionally, the water gradient‐induced ions migration is visually approved by the acid indicator composed of bromophenol blue and isopropyl alcohol (Figure S33, Supporting Information). This indicator has a color transformation from blue to yellow when the pH value is changed due to the increased protons by adding acid chemicals.^[^ ^19^ ^]^ As shown in Figure 5g, the bromophenol blue‐based acid indicator has initial blue colors on both sides of hydrogels. By dropping 0.5 mL water into the upper bromophenol blue, the color of the region above the hydrogel film is changed into yellow, demonstrating the dissociation of free protons from the hydrogel. Further diffusion of protons and water to the bottom tube leads to the initiation of color change in the bottom until the color of the acid indicator solution totally turns yellow. The above process mimics the ion transport in the hydrogel, showing that the high output of the MEG device originates from the continuous ion diffusion driven by concentration difference.

**Figure 6:**
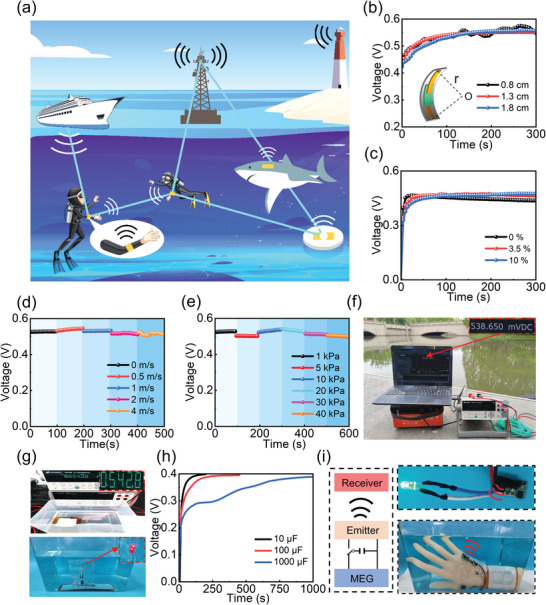
Integrated MEGs for self‐powered wearable applications underwater. a) Schematic illustration of MEGs for signal transmission network underwater. The vector graphics in the figure are sourced from the Vecteezy website, which provides free licensed images. b) The output of the MEG device at different bending angles with an inset showing schematic diagrams of various bending angles. Output of the MEG at different c) salt concentrations, d) disturbing flow speeds, and e) water depths. f) The robust voltage output of a device output under a natural wild river with a depth of 5 m. g) Top: Voltage output of a single device underwater. Bottom: 10 connected MEG devices underwater capable of lighting an LED after charging a capacitor. h) Charging process of commercial capacitors connected with a single MEG device. i) Left: Scheme of wireless remote communication system powered by the electricity from MEGs in water. Right: (bottom) Integrated wearable MEG devices charging a 470 µF capacitor to trigger a wireless emitter in water and (top) the emitter transmitting information to a remote receiver for optical alarming.

We now finally demonstrate experimentally that the electricity generated from our MEG device can power electronic devices in water for remote wireless communications (**Figure** **6**a). Such a self‐powered wearable system can be potentially used as a wireless tracker for sending immediate location information to a monitor underwater, providing a facile approach for location tracking in urgent situations. To demonstrate this capability, we first bend the device to test its electronic output. The output voltage maintains a stable value of 0.55 V with a bending radius from 0.8 to 1.8 cm (Figure 6b), demonstrating a stable power supply for wearable electronics underwater. As real underwater conditions present salts, we then place the device in highly salty water to check the output stability. Figure 6c shows that the device has a very robust and stable output, even though the salt concentration is as high as 10% already largely exceeds most water sources on Earth. The exceptional stability is attributed to the presence of the E‐PTFE layer only allowing water vapor to transmit and blocking any liquid with salts dissolved. We next check the output stability underwater at different water‐flow speeds and hydraulic pressures. As the E‐PTFE layer can effectively protect the hydrogel device from the liquid water, even the 1 m s^−1^ disturbing flow does not influence the electricity output (Figure 6d). Due to the constant and stably high RH induced by water vapor penetration through the E‐PTFE layer (Figure S15, Supporting Information), the device can yield a stable voltage of 0.55 V under hydraulic pressures ranging from 1 to 40 kPa (Figure 6e; Figure S34, Supporting Information), which demonstrates the outstanding underwater performance. The above results indicate that our MEG device has excellent operational stability even in harsh underwater environments. The picture of the MEG device under a natural wild river with a depth of 5 m with a robust electricity output can be seen in Figure 6f.

As the integration of multiple MEG devices to amplify the electrical output is essential for practical underwater applications, we then connect the devices in series or parallel to increase the voltage or current, respectively. The first‐to‐end electrode connection was assembled to create large‐area integration, then a large E‐PTFE layer was used to seal the entire assembly to protect it from liquid water (Figure S35, Supporting Information). The size and the total number of MEG units can be easily tailored according to the practical application requirements. As shown in Figure S36 (Supporting Information), by connecting 10 units in series, an output voltage of up to 4 V can be achieved. Similarly, an output current can reach 0.9 mA by integrating 10 units in parallel, demonstrating good scale‐up ability. As shown in Figure 5g, the electricity generated from integrated units can be used to power LED underwater, which is bright enough to be seen in the daytime. In addition, our MEG can charge commercial capacitors ranging from 10 to 1000 µF rapidly (Figure 6h), which could be used for high‐power applications.

Based on the above excellent operationalities, an underwater wireless system with MEG for remote communication is demonstrated. An integration of 10 MEG devices connected in series was placed into a chamber filled with water. A low‐power wireless emitter (power consumption is ≈10 mW), together with a commercial capacitor with 470 µF was sealed into a waterproof bag. The capacitor was first charged with the MEG assembly. As shown in Figure S37 (Supporting Information), the capacitor can be charged up to 2.4 V in 2000 s. Then, the wireless emitter was driven by the capacitor to generate RF signals underwater for remote communication. The scheme is illustrated in the left panel of Figure 6i. A proof‐of‐the‐concept of the remote emergency alarm system with under underwater self‐powered wearable wireless communication circuit is shown in the right panel of Figure 6i. Once the remote router acquires the signal from the underwater emitter powered by the MEG, the alarming LED starts to flash (Movie S7, Supporting Information). Different from conventional devices, this new type of self‐powered underwater wireless communication system does not require a battery, which holds great promise for location tracking for a wide range of applications such as human diving,^[^ ^43^ ^]^ marine animal inhabitation,^[^ ^44^ ^]^ and marine environments monitoring.^[^ ^45^ ^]^

## 3 Conclusion

In summary, we develop a biodegradable, self‐healable, flexible, and scalable MEG device that can directly harvest energy from moisture in the underwater environment. This device is based on a sandwiched molecular‐engineered hydrogel sealed with a waterproof breathable membrane made from E‐PTFE allowing water vapor to be transferred while preventing liquid water penetration. The MEG shows excellent self‐healing ability, biocompatibility, and flexibility. Underwater environment, the device can spontaneously and continuously deliver a voltage of 0.55 V and a current density of 130 µA cm^−2^, which is due to the efficient ion separation assisted by negative ions confinement in hydrogel networks. The electricity output is maintained with 10% salt concentration, 1 m s^−1^ disturbing flow, as well as >40 kPa hydraulic pressure, exhibiting outstanding harsh environment tolerance underwater. As the first demonstration of practical applications using MEG in self‐powered underwater electronics, this new type of device is successfully powering emitter in water for location tracking and wireless remote alarming. This work offers an innovative route for harvesting moisture energy underwater, suggesting our complementary device holds promise in the creation of a new range of innovative electronic devices for marine Internet‐of‐Things.

## 4 Experimental Section

### Materials

Polyglutamic acid (PGA, *M*_n_ 1 00 000–2 00 000) was purchased from Henan Jujin Bio‐Tech Co., Ltd. Citric acid (CA), sodium chloride granules (NaCl), and isopropanol/bromophenol blue indicator (0.1% w/v) were purchased from Aladdin Limited. E‐PTFE membrane is obtained from Dongguan Jinlong Net Industry Co., Ltd. Graphite paper, gold foil, carbon tape, conductive copper tape, silver foil, conductive silver paste, and epoxy were purchased at the Taobao online shop. All reagents were used without further processing.

### Device Preparation

PGA powder (0.5 g) and deionized water (10 g) were stirred for 1 h under a magnetic stirrer, followed by 30 min of sonication to prepare a uniform 5% PGA solution. Simultaneously, 1 g of CA was added to 10 g of deionized water which was stirred thoroughly to form a 10% CA solution. The solution with different ratios of PGA and CA was stirred for 30 min followed by 30 min sonication to form mixed solutions with different concentrations. The mixed solution was then drop‐casted onto electrodes and dried for 12 h at 25 °C and 30% humidity to obtain PGA‐CA ionic hydrogel membrane. The 20 µm thick film can be obtained from 0.25 mL solution after drying. Thus, the thickness can be adjusted by dropping different volumes of mixed solution. After drying, a top electrode foil with holes was attached to the upper surface of the PGA–CA membrane. Finally, the E‐PTFE membrane was overlaid on the device, and the device was sealed to prevent direct contact with liquid water.

### Materials Characterization

The absorption dynamic process of the ionic hydrogel under 80% humidity conditions was analyzed by acquiring the mapping feature using a Raman spectrometer (Thermo Fisher DXR Raman spectrometer, laser 633 nm). The hydrophobicity of hydrogel films and E‐PTFE films was measured using a contact angle measurement instrument (KRUSS GmbH, DSA30). The self‐healing process of ionic hydrogel films and the thickness of E‐PTFE film were observed under an optical microscope (Olympus). The microstructure of the E‐PTFE film was characterized using a scanning electron microscope (SEM, Japan Hitachi S‐3400N). Kelvin probe force microscopy (KPFM) was used to measure the potential on both sides of the film through an atomic force microscope (Bruker, Multimode 8). The chemical functional groups of ionic hydrogel films were analyzed using Fourier‐transform infrared spectroscopy (PerkinElmer Spectrum 100) under attenuated total reflection (ATR) mode. The elemental composition was analyzed using X‐ray photoelectron spectroscopy (ESCALAB Xi+ Spectrometer).

### Electrical Measurement

The relative humidity in the chamber was controlled by flowing gas generated from different saturated salt solutions. Simultaneously, a humidity meter (FY‐12, Shenzhen Dreamberry Electronic Technology Co., Ltd.) was employed to monitor the relative humidity in the chamber. For underwater signal acquisition, the encapsulated MEG device is directly immersed in water. The water pressure is regulated by the position of the MEG device in the experimental bucket containing water. The MEG device was placed in a deeper pool of ship model to improve the water pressure. A hygrometer (FY‐12) was used to detect the humidity changes inside the MEG device under different water pressure. The top and bottom electrodes of the MEG device were directly connected to the Agilent 34410A digital multimeter under high‐z operation mode for signal output. In the actual environment, the MEG device is sunk into the lake by counterweight, and the wire of the MEG device is connected to the Agilent 34410A digital multimeter to measure the electrical signal.

## Conflict of Interest

The authors declare no conflict of interest.

## Supporting information



Supporting Information

Supplemental Movie 1

Supplemental Movie 2

Supplemental Movie 3

Supplemental Movie 4

Supplemental Movie 5

Supplemental Movie 6

Supplemental Movie 7

## Data Availability

The data that support the findings of this study are available from the corresponding author upon reasonable request.
