# Supplementary material for: Ionic Hydrogel‐Based Moisture Electric Generators for Underwater Electronics
Source: Adv Sci (Weinh). 2024 Sep 29;11(43):2408954. doi: 10.1002/advs.202408954 (PMC11578371; doi:10.1002/advs.202408954)
Supplement: Supplementary file 1 — Supporting Information [file ADVS-11-2408954-s006.pdf]

ADVANCED SCIENCE
Open Access

Supporting Information

for *Adv. Sci.*, DOI 10.1002/advs.202408954

Ionic Hydrogel-Based Moisture Electric Generators for Underwater Electronics

Daozhi Shen, Fangzhou Li, Jian Zhao*, Rui Wang*, Bin Li, Zechao Han, Linglan Guo, Peicheng Han, Dongqi Yang, Hyun Ho Kim, Yanjie Su, Zhixiong Gong and Limin Zhu*

Supplementary information

Ionic Hydrogel-based Moisture Electric Generators for Underwater Electronics

Daozhi Shen [a, b, #, *], Fangzhou Li [c, #], Jian Zhao [c, *], Rui Wang [d, *], Bin Li [c], Zechao Han [a], Linglan Guo [a], Peicheng Han [a], Dongqi Yang [a], Hyun Ho Kim [e], Yanjie Su [f], Zhixiong Gong [g], Limin Zhu [a,h]

[a] School of Mechanical Engineering, Shanghai Jiao Tong University, Shanghai 200240, China

[b] National Center for Translational Medicine, Shanghai Jiao Tong University, Shanghai 200240, China

[c] School of Materials Engineering, Shanghai University of Engineering Science, Shanghai 201620, China

[d] University of Michigan - Shanghai Jiao Tong University Joint Institute, Shanghai Jiao Tong University, Shanghai 200240, China

[e] Department of Energy Engineering Convergence, School of Materials Science and Engineering, Kumoh National Institute of Technology, Gumi39177 Republic of Korea

[f] Department of Micro/Nano Electronics, School of Electronics Information and Electrical Engineering, Shanghai Jiao Tong University, Shanghai 200240, China

[g] State Key Laboratory of Ocean Engineering, School of Ocean and Civil Engineering, Shanghai Jiao Tong University, Shanghai, 200240, China

[h] State Key Laboratory of Mechanical System and Vibration, Shanghai Jiao Tong University, Shanghai 200240, China

Email: dzshen@sjtu.edu.cn; 05160001@sues.edu.cn; rui.wang@sjtu.edu.cn

Simulation methods

All density functional theory (DFT) calculations were performed by VASP (Vienna ad initio simulation package) [1-3]. The electronic exchange and correlation interaction were described by Per-dew-Burke-Ernzerhof (PBE) parameterization of general gradient approximation (GGA) [4]. The cutoff energy of plane wave expansion was set to be 400 eV. The optimization was terminated if the total energy difference is below 10^{-5} eV and the maximum force of each atom was less than 0.01 eV/Å. The formation energy was calculated as $E = E_{tot} - E_{ploy} - E_{wt}$, where E_{tot}, E_{ploy} and E_{wt} were the total energy of the system, the energy of PGA (or PGA-CA), and the energy of H_2O, respectively. The quantitative analysis of electrostatic surface potential (ESP) on vdW molecular surface was calculated by Multiwfn 3.8 program [5,6]. The color mapped isosurface graphs of ESP were rendered by Visual Molecular Dynamics (VMD) 1.9.4a51 program [7]. The ab initio molecular dynamics (AIMD) simulations were performed using the canonical ensemble at 300 K for 2ps with a time step of 2 fs to simulate a thermal vibration environment. The density of all initial configurations was obtained from classical molecular dynamic (MD) simulation.

The classical MD simulations were performed using the LAMMPS (the large-scale atomic/molecular massively parallel simulator) simulation package [8]. The polymer consistent force field (pcff+) developed by MedeA [9,10] was employed to describe intramolecular interactions and partial charges. Non-bonded interactions were modeled using COMPASS (class 2) [11] with long-range Coulombic interactions handled by a particle-particle particle-mesh solver [12]. A Nose–Hoover thermostat [13] was employed to control the temperature at 300 K and the pressure at 1 bar. The velocity Verlet algorithm with a time step Δt = 0.05 fs was applied, and the total length of each simulation was 10 ns. The coordinates of atoms were recorded every 10 ps for post-processing analyses.

Diffusion coefficients are computed using the mean square displacement (MSD) [1]:

$$MSD = \langle |r(t) - r(0)|^2 \rangle$$

$$D = \frac{1}{6} \lim_{\Delta t \to \infty} \frac{MSD(t + \Delta t) - MSD(t)}{\Delta t}$$

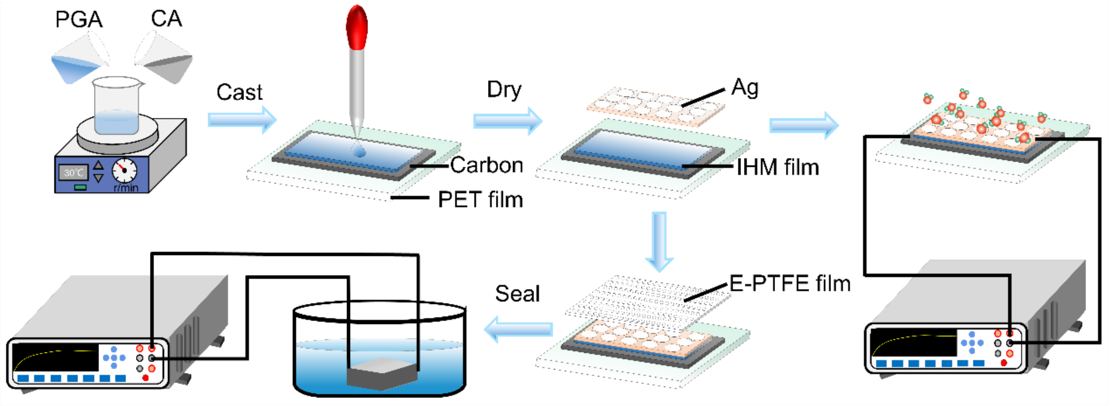

Figure S1 The preparation process and electrical measurement of MEG devices.

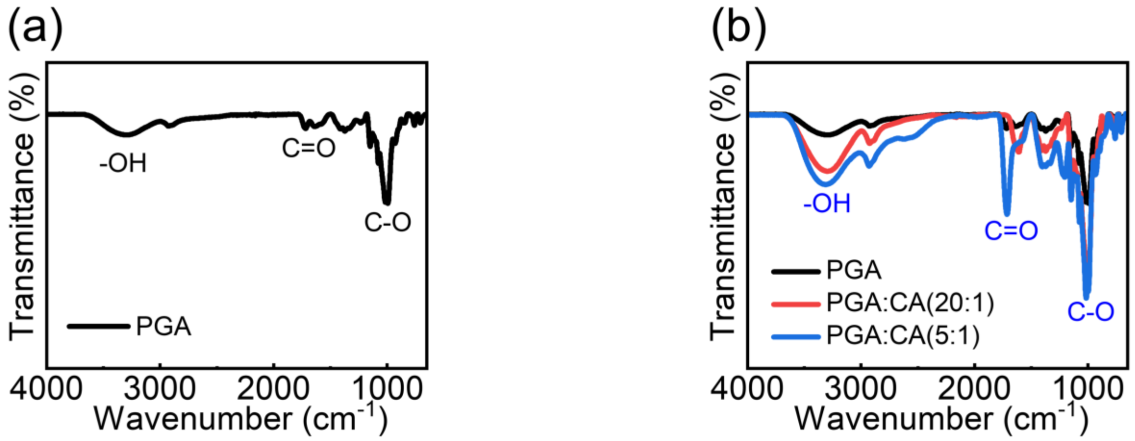

Figure S2 FTIR spectra of films. (a) FTIR spectra of PGA. (b) Comparison of FTIR spectra of PGA, PGA: CA (20:1) and PGA: CA (5:1).

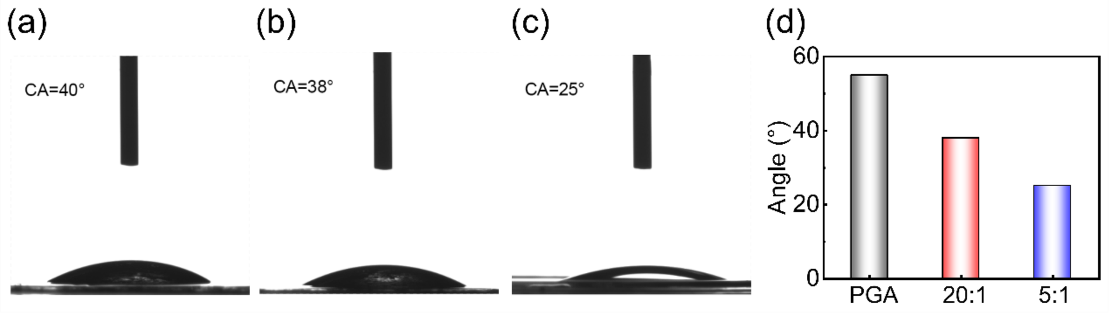

Figure S3 Water contact angle measurement of active materials. (a) PGA. Mixing ratio of (b) 20:1 and (c) 5:1. (d) Contact angles summary.

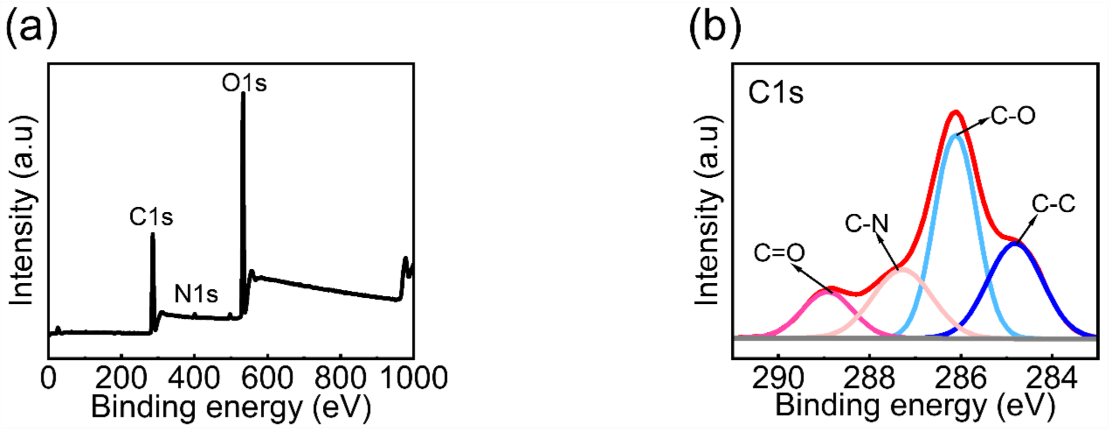

Figure S4 XPS photoelectron spectra of PGA-CA hydrogel film with PGA to CA ratio of 5:1. (a) XPS full spectrum. (b) The intensity of C1s in the XPS spectrum, which contains a large number of C-O and C=O bond -COOH, greatly improves the water absorption of the film.

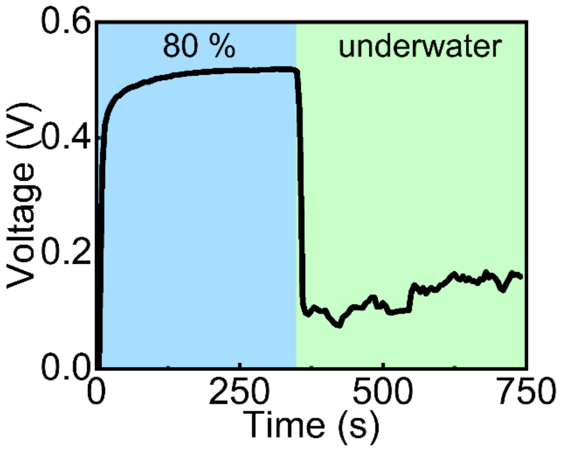

Figure S5 Output voltage of a MEG device without E-PTFE membrane when immersed into liquid water.

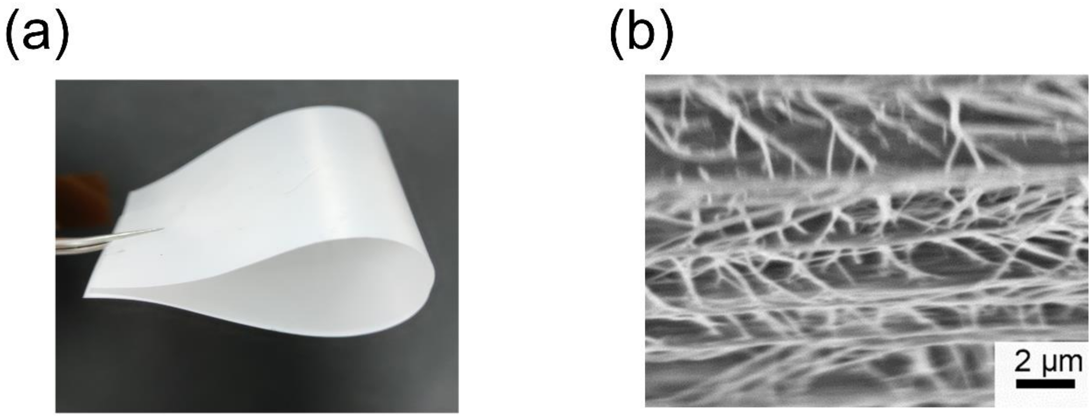

Figure S6 E-PTFE film characterization. (a) Photo of E-PTFE film with flexibility. (b) SEM image of E-PTFE film.

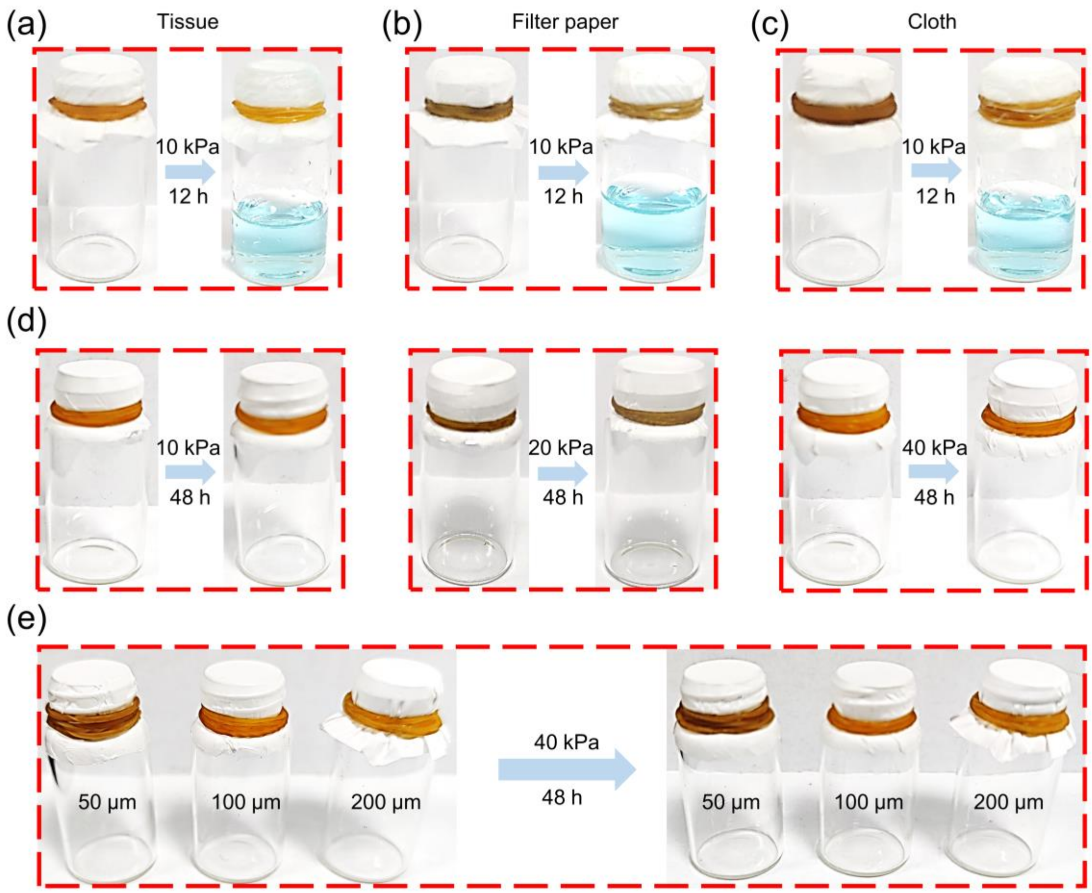

Figure S7 Liquid water leakage test of different membranes. (a) Glass bottles initially encapsulated with (a) regular tissue, (b) filter paper, and (c) cloth are immersed into water at 10 kPa for 12 h, showing obvious leakage. (d) Glass bottle initially encapsulated with 100 μm thick E-PTFE film is immersed into liquid water at 10 kPa, 20 kPa and 40 kPa for 48 h, showing no leakage. (e) Glass bottle initially encapsulated with E-PTFE films with different thickness is immersed into liquid water at 40 kPa for 48 h, showing no leakage.

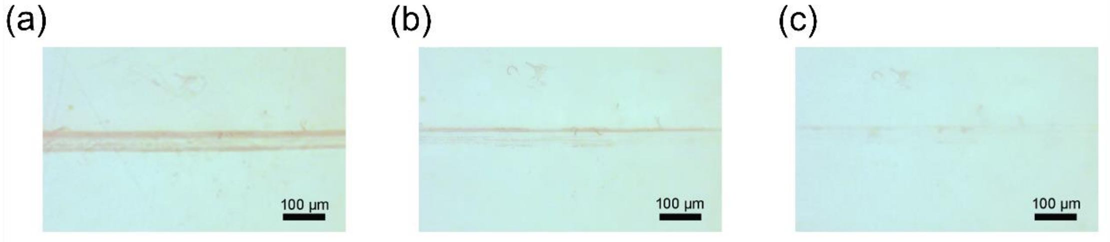

Figure S8 The self-healing process of PGA-CA film at 90% humidity. (a) Pristine scratch cutting. (b) Healing at 30 minutes. (c) Healing at 60 minutes.

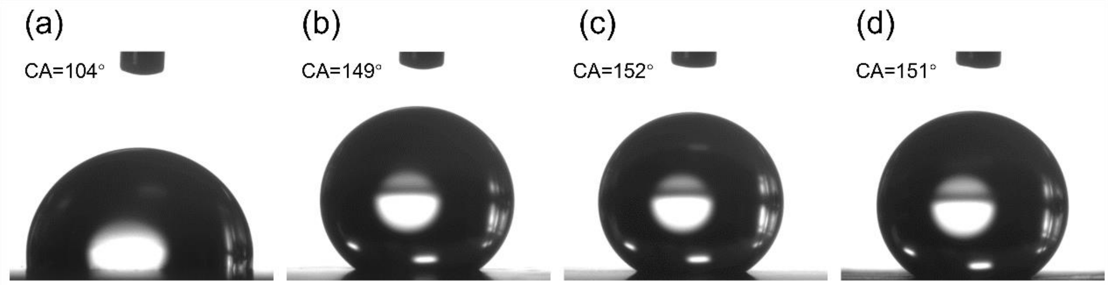

Figure S9 Water contact angle measurement of E-PTFE with different pore sizes conditions. (a) No pores. Pore diameter is (b) 0.1 μm, (c) 0.3 μm, and (d) 1 μm.

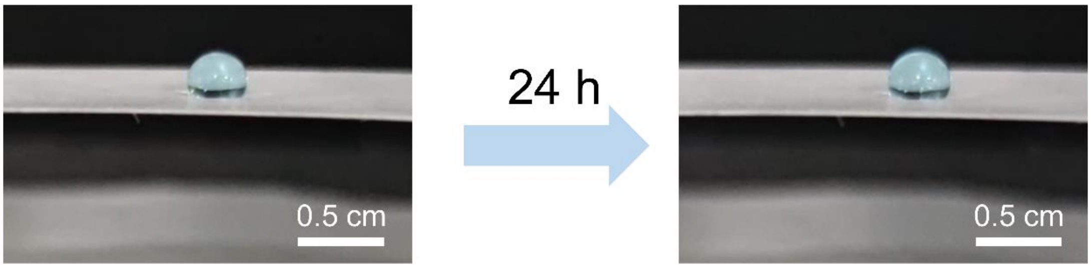

Figure S10 A water drop was placed on a E-PTFE waterproof breathable membrane. No liquid penetration is abvious after 24 h.

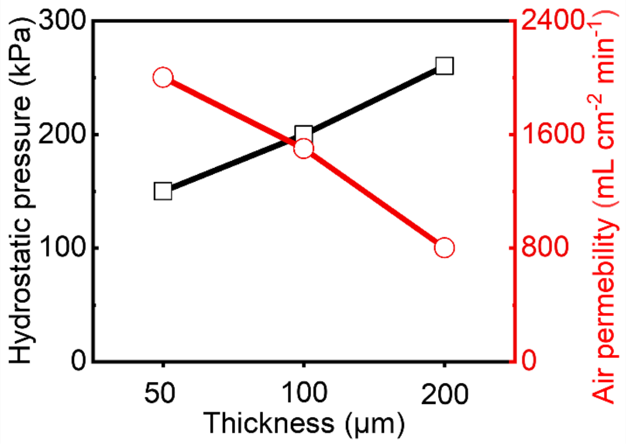

Figure S11 The hydrostatic pressure limit and air permeability of E-PTFE waterproof breathable membrane vs. film thickness.

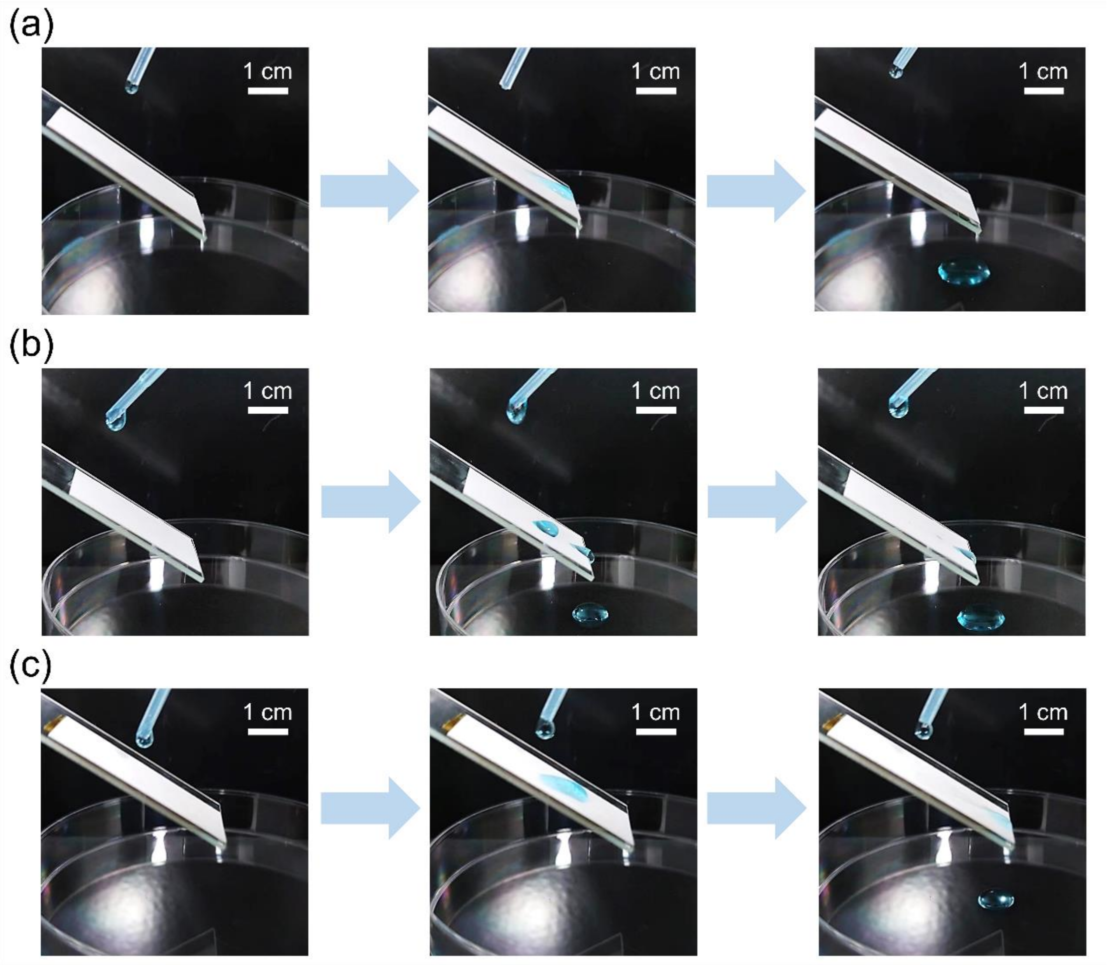

Figure S12 Optical image of a water drop impacting on porous E-PTFE film with different pore sizes. The pore size is (a) 0.1 μm, (b) 0.3 μm, and (c) 1 μm.

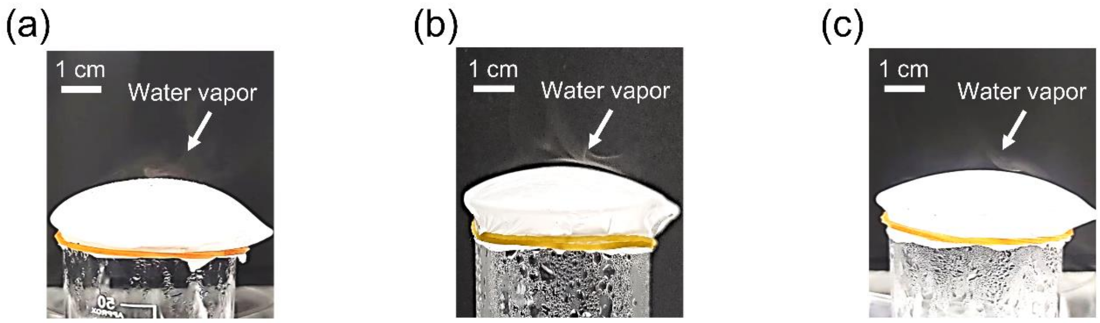

Figure S13 Water vapor permeability performance of porous E-PTFE film with different pore sizes. The pore size is (a) 0.1 μm, (b) 0.3 μm, and (c) 1 μm.

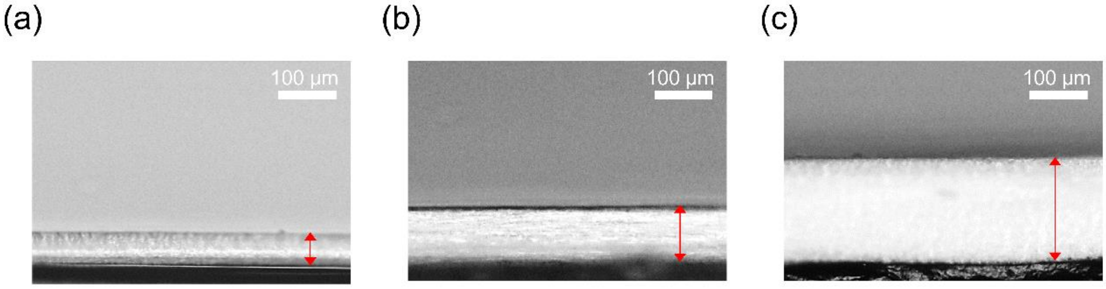

Figure S14 Optical cross-sectional images of porous E-PTFE film with different thickness. The thickness is (a) 50 μm, (b) 100 μm, and (c) 200 μm.

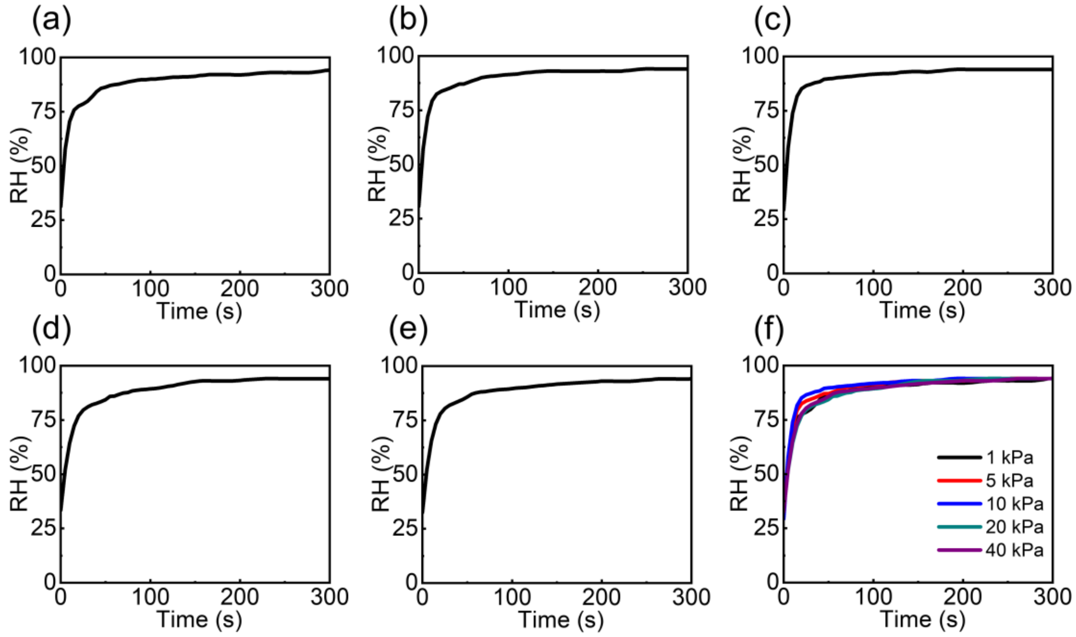

Figure S15 The change of RH in a chamber sealed with E-PTFE film in liquid water under different hydrostatic pressures. The pressure is (a) 1 kPa, (b) 5 kPa, (c) 10 kPa, (d) 20 kPa, and (e)40 kPa. (f) Humidity changes summary.

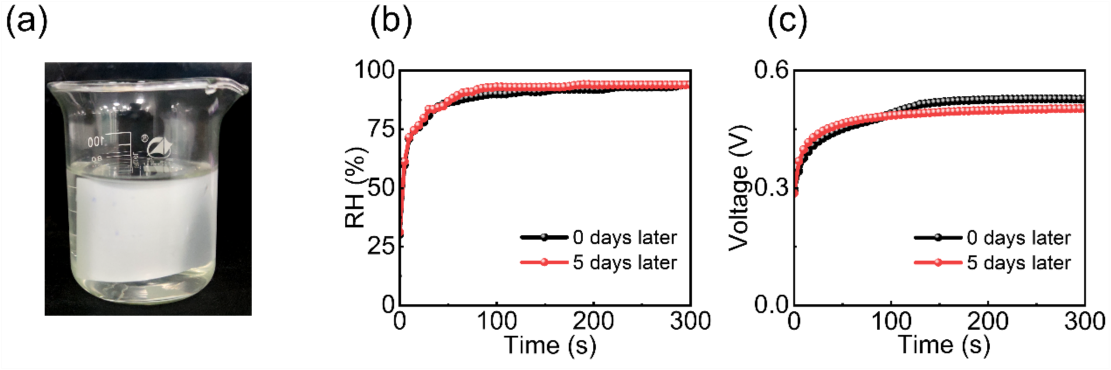

Figure S16 The performance of a E-PTFE film immersed in water for 5 days. (a) Optical image of E-PTFE film immersed in water. The comparison of (b) water vapor transmission efficiency indicated by RH change and (c) electricity output capability of E-PTFE film after contact with water for 5 days, showing stable performance.

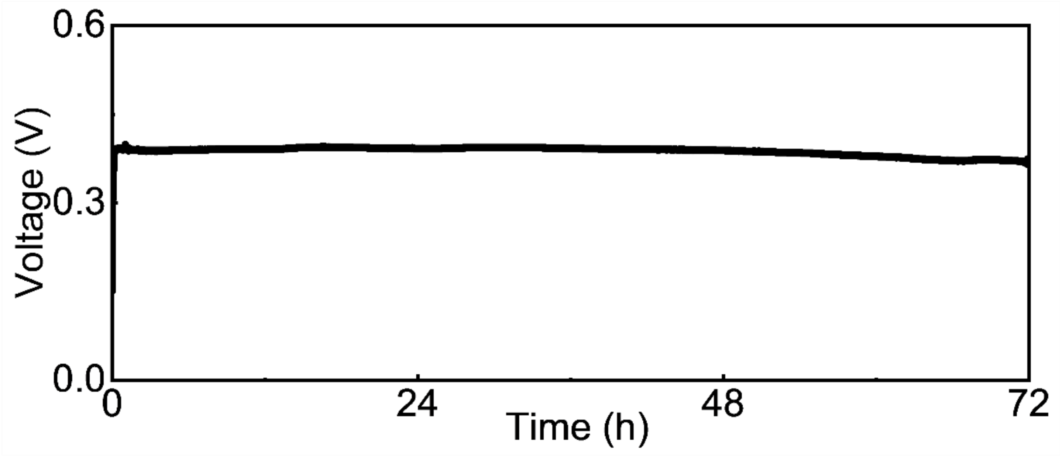

Figure S17 Long term output characteristic of MEG device in wet environment.

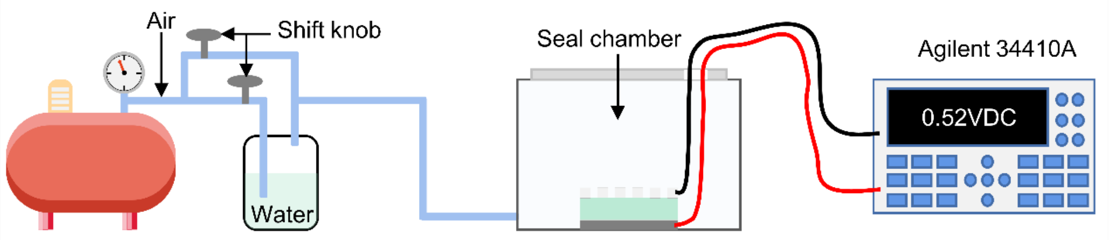

Figure S18 Experimental setup of the power generation process.

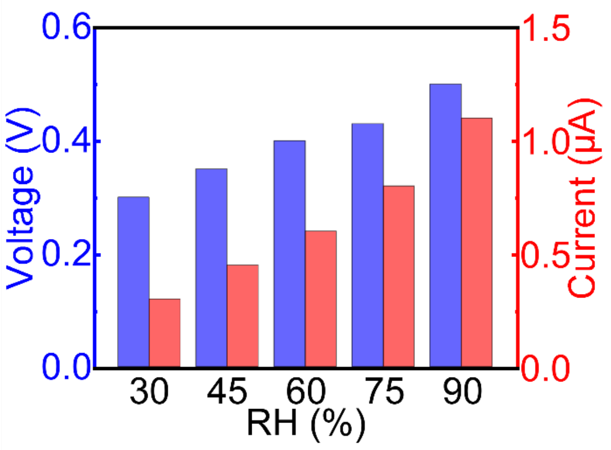

Figure S19 Output of MEG under different RHs.

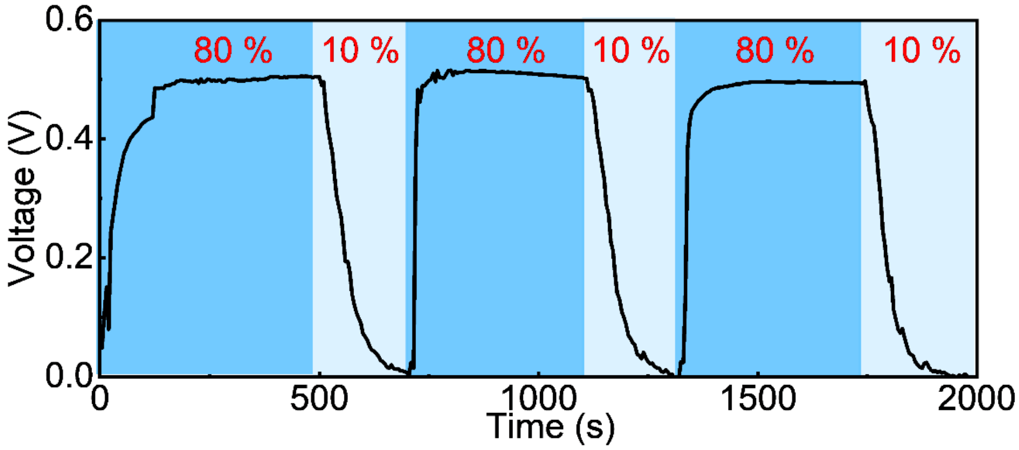

Figure S20 Voltage output of MEG under cyclic RH changing.

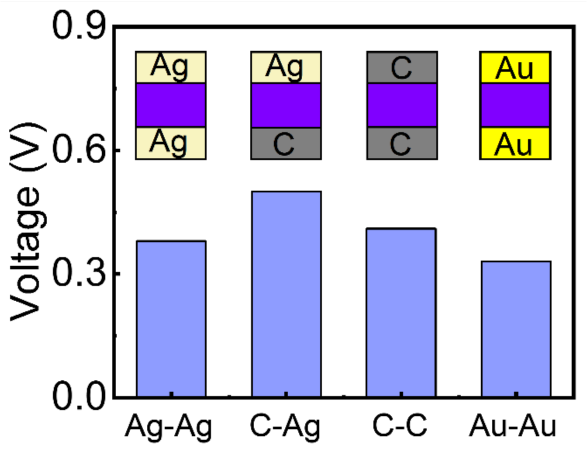

Figure S21 Voltage output of MEG devices with different inert electrodes at RH of 90%.

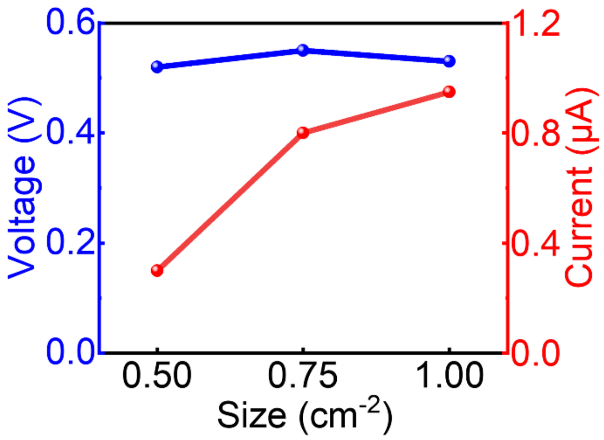

Figure S22 Output of MEGs with different areas.

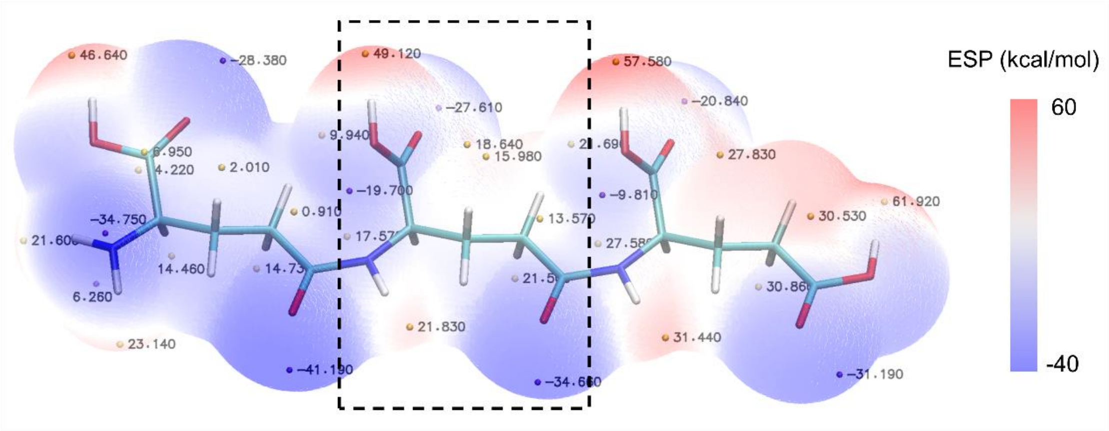

Figure S23 Minima and maxima of ESP on the vdW surface for PGA with their values. The single PGA cell for periodic boundary conditions is marked with dashed rectangle.

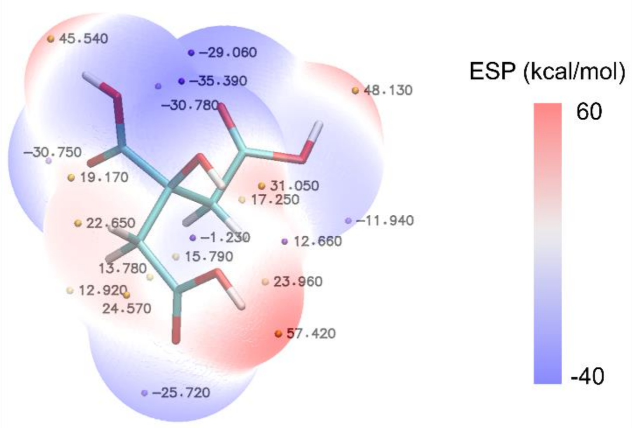

Figure S24 Minima and maxima of ESP on the vdW surface for CA with their values.

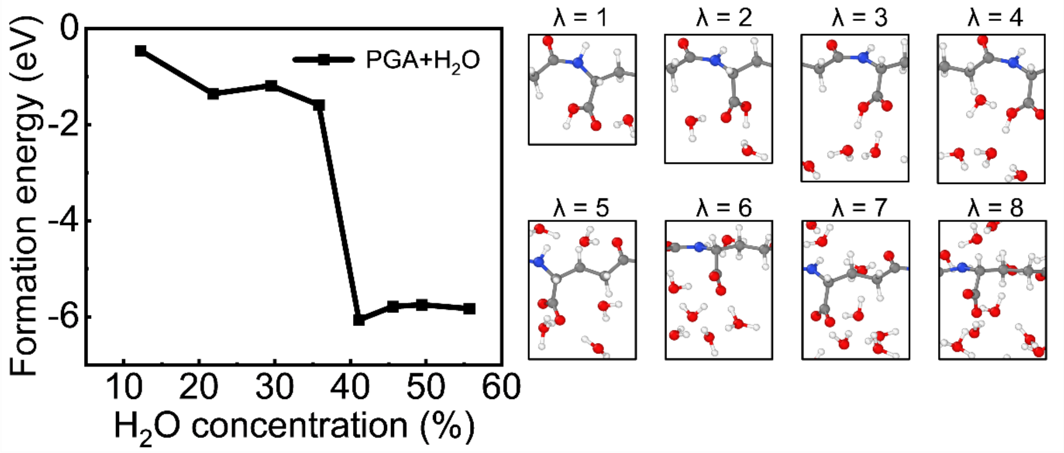

Figure S25 Formation energy with snapshots for PGA-water system. The water concentration increases with λ = n(H2O) / n(PGA unit) from 1 to 9. The four snapshots on the top are the systems of water concentration lower than 0.4 (λ = 1 ~ 4), with no protons dissociation. The four snapshots at bottom are the systems of water concentration higher than 0.4 (λ=5~9), with protons dissociation from -COOH group.

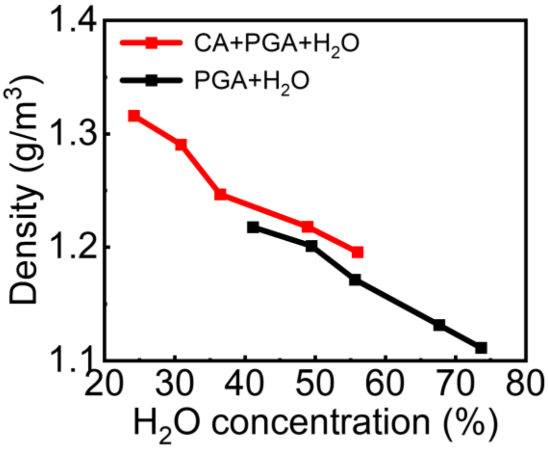

Figure S26 The density of the mixed system vs water concentration.

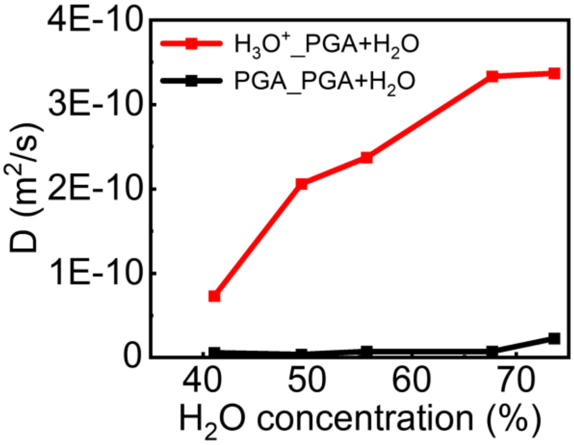

Figure S27 The diffusion coefficients of proton and PGA vs water concentration.

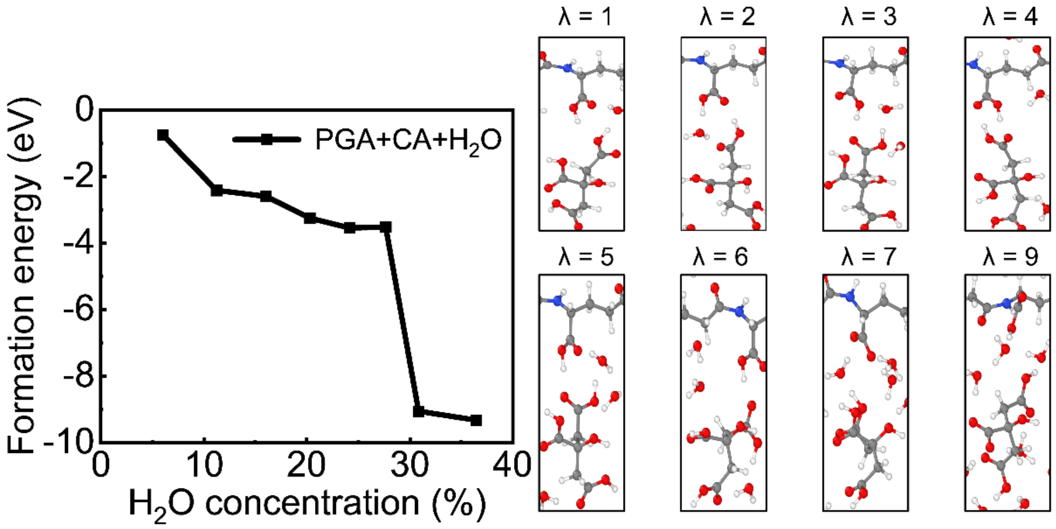

Figure S28 Formation energy with snapshots for PGA-CA-water system. The six snapshots on the top are the systems of water concentration lower than 0.27 (λ = 1 ~ 6), with no protons dissociation. The two snapshots at bottom are the systems of water concentration higher than 0.27 (λ=7~9), with protons dissociation from -COOH groups of PGA-CA.

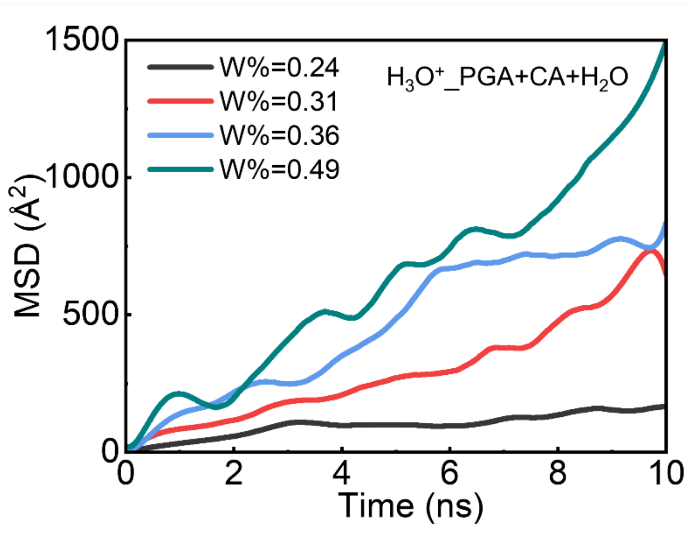

Figure S29 MSD of the PGA-CA-water system with time at different water concentrations.

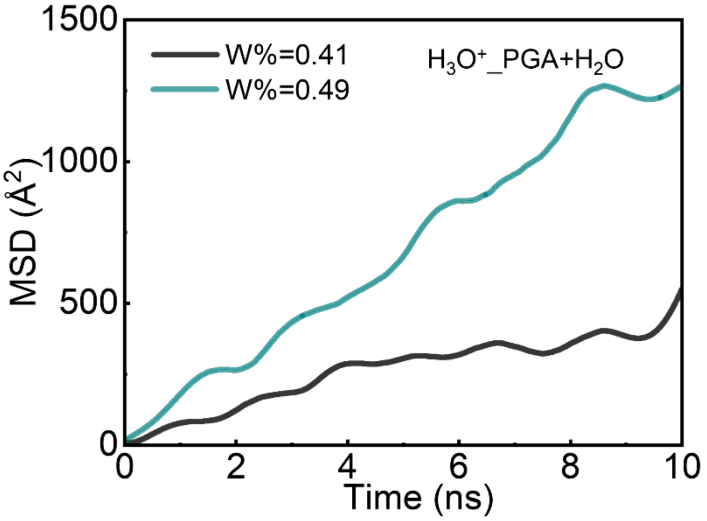

Figure S30 MSD of the PGA-water system with time at different water concentrations.

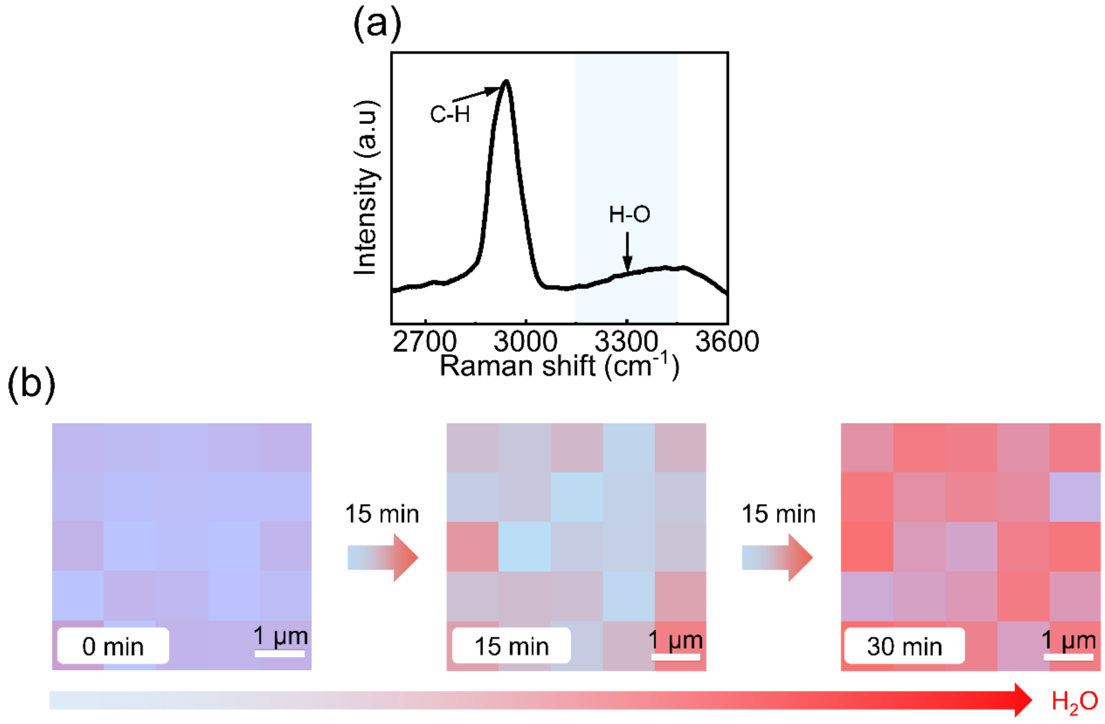

Figure S31 Raman Characterization of PGA-CA. (a) Raman spectra of the PGA-CA film surface, where arrows point to the integral intensities of the C-H and O-H bond. (b) Water absorption in a PGA-CA film under moist environment using Raman mapping spectroscopy.

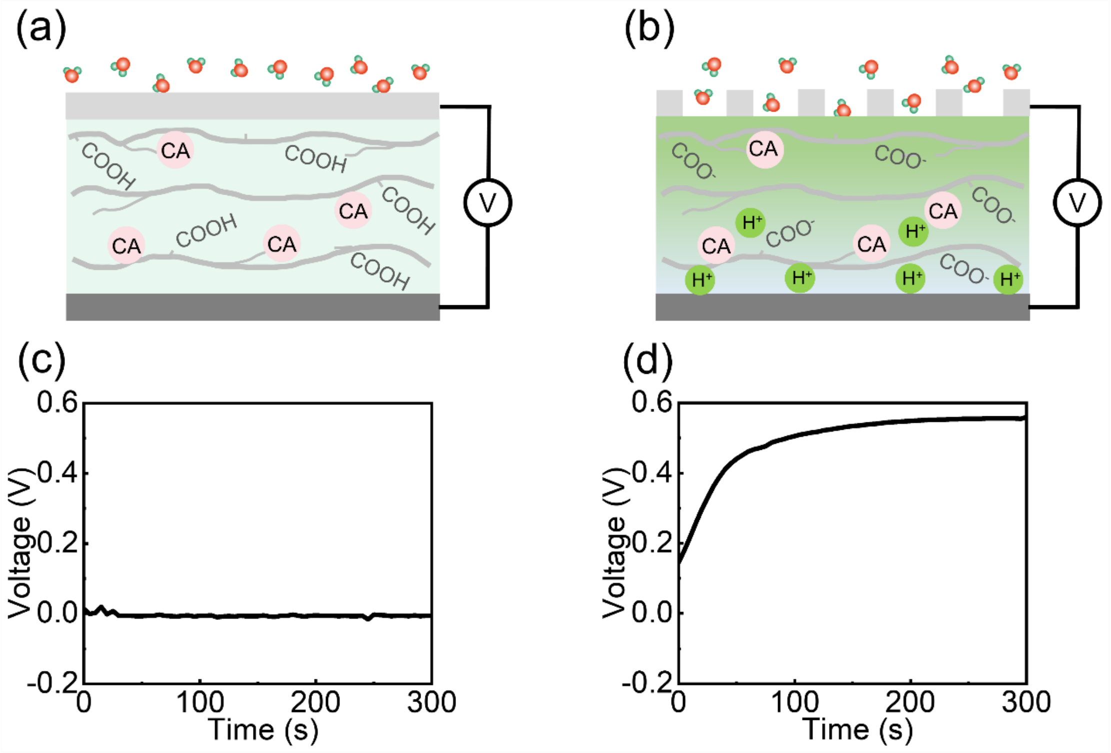

Figure S32 Output of device with and without holes in top electrodes. (a) Schematic diagram of MEG devices with no hole in the upper electrode. (b) Schematic diagram of a MEG devices with holes in the upper electrode. (c) Voltage output curve corresponding to device in (a). (d) Voltage output curve of the device in (b).

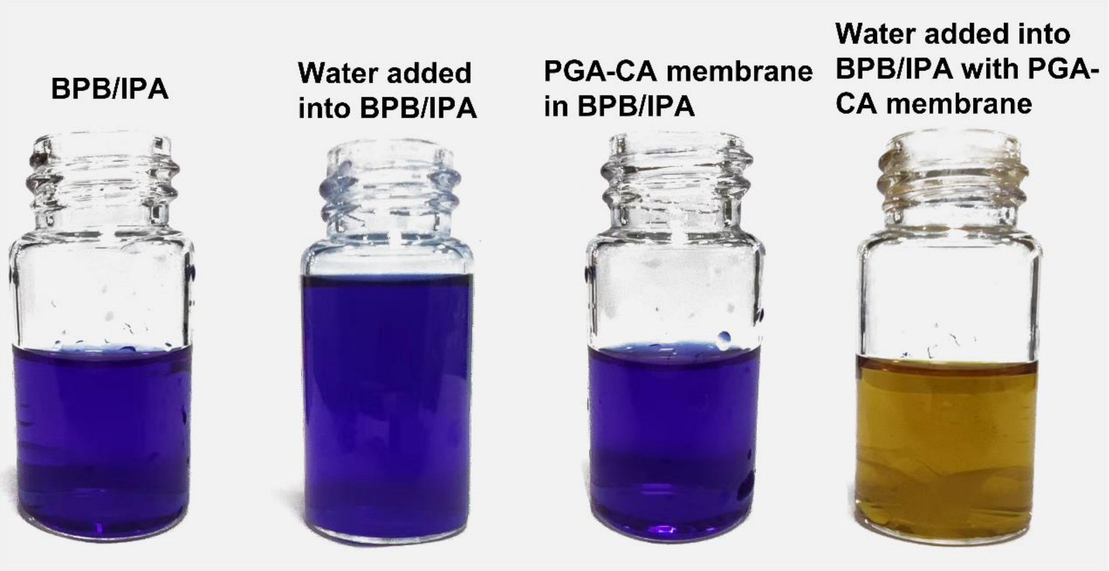

Figure S33 Photos of BPB/IPA indicators under different conditions. From left to right: Bromophenol blue (BPB) and isopropyl alcohol (IPA) solutions; Only bromophenol blue (BPB) and isopropyl alcohol (IPA) are added to water; Only PGA-CA film is added to bromophenol blue (BPB) and isopropyl alcohol (IPA) solution; Water and PGA-CA film are added to bromophenol blue (BPB) and isopropyl alcohol (IPA) solutions. The results show that water dissociation PGA-CA film is the main cause of H^+ release.

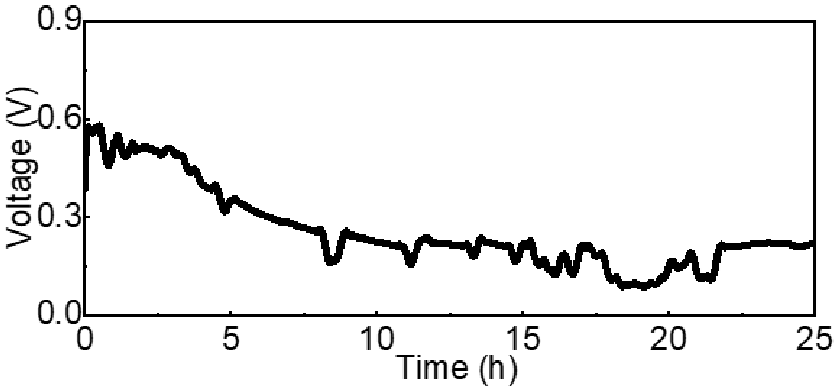

Figure S34 Electrical output of MEG devices underwater.

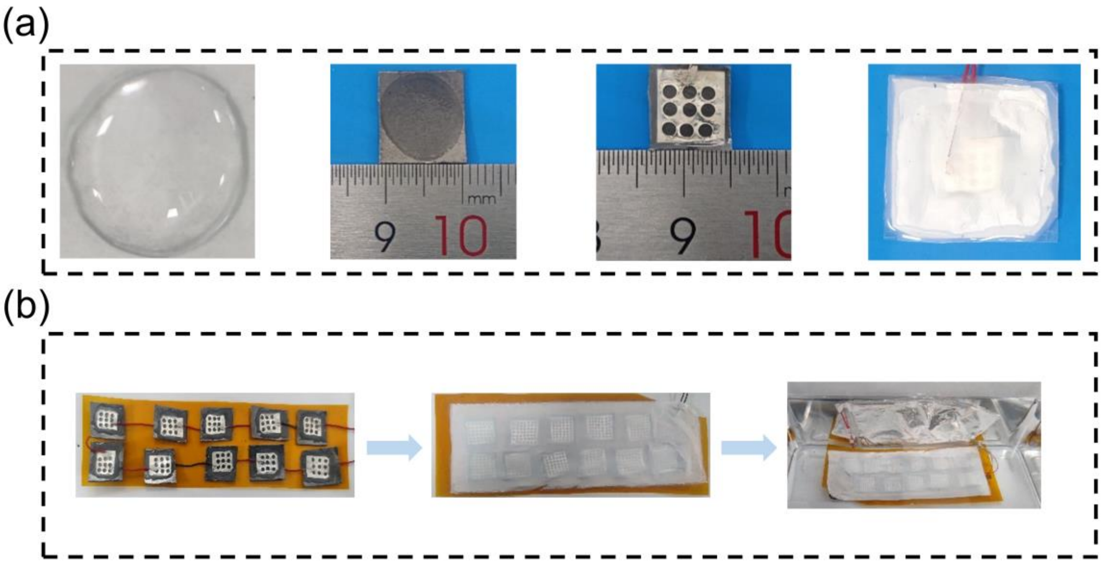

Figure S35 Photos of PGA-CA and MEGs. (a) Photos of transparent hydrogel and the device. (b) Photos of 10 MEG devices in series before and after E-PTFE covering.

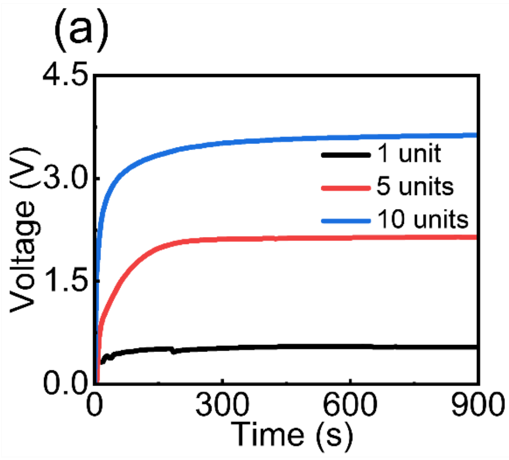

Figure S36 Electric output of integrated MEGs. (a) Voltage output of 1, 5 and 10 MEG devices connected in series. (b) Current discharging of 1, 5 and 10 MEG devices connected in parallel.

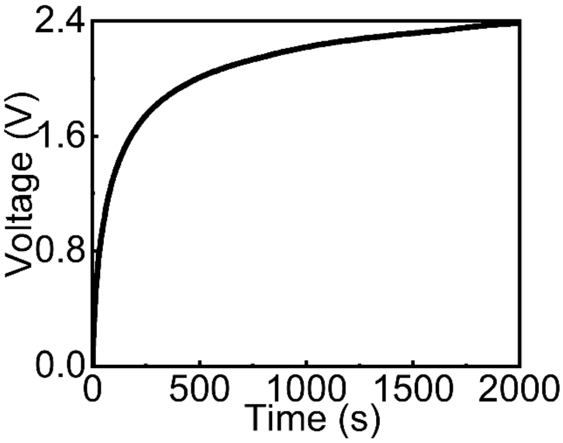

Figure S37 Charge process of a 470 μF capacitor connected with 10 MEG devices in liquid water to trigger a wireless transmitter.

Supplementary Table S1

**Material**	**RH (%)**	**Voltage (V)**	**Current (μA·cm^{-2})**	**Underwater work**	**Biocompatible**	**Stability**	**Ref**
graphene oxide	70	0.45	0.2	No	No	continuous	14
TiO_2	85	0.5	9	No	No	transient	15
GO–grGO	80	1.5	0.15	No	No	transient	16
polymer nanowires	75	0.15	0.4	No	No	transient	17
graphene oxide	80	0.6	1.2	No	No	continuous	18
biologic nanofibrils	99	0.12	0.05	No	yes	continuous	19
protein nanowires	60	0.5	17	No	yes	continuous	20
Geobacter sulfurreducens	75	0.33	0.5	No	yes	continuous	21
MoS2 nanosheets	80	0.02	6	No	No	continuous	22
polyelectrolyte films	85	1.38	0.9	No	yes	continuous	23
Nb2CTx/sodium alginate	90	0.5	1.6	No	No	transient	24
SAG film	100	0.5	100	No	No	continuous	25
Polymer film	80	1.1	3	No	No	continuous	26
PVA–PNIPAM Hydrogel	80	0.34	24	No	yes	continuous	27
SA-GO fiber	70	0.25	4	No	No	continuous	28
PGA-CA film	90	0.55	1.2	yes	yes	continuous	This work

References

[1] G. Kresse, J. Hafner, *Physical Review B* **1994**, *49*, 14251.
[2] G. Kresse, J. Furthmüller, *Physical Review B* **1996**, *54*, 11169.
[3] G. Kresse, J. Furthmüller, *Computational Materials Science* **1996**, *6*, 15.
[4] J. P. Perdew, K. Burke, M. Ernzerhof, *Physical Review Letters* **1996**, *77*, 3865.
[5] T. Lu, F. Chen, *Journal of Computational Chemistry* **2012**, *33*, 580.
[6] J. Zhang, T. Lu, *Physical Chemistry Chemical Physics* **2021**, *23*, 20323.
[7] W. Humphrey, A. Dalke, K. Schulten, *Journal of Molecular Graphics* **1996**, *14*, 33.
[8] S. Plimpton, *Journal of Computational Physics* **1995**, *117*, 1.
[9] M. Parrinello, A. Rahman, *Journal of Applied Physics* **1981**, *52*, 7182.
[10] Materials Design, I. Medea-Materials Exploration and Design Analysis: San Diego, CA, USA, **2018.**
[11] H. Sun, *Journal of Physical Chemistry B* **1998**, *102*, 7338.
[12] Hockney and Eastwood, Computer Simulation Using Particles, Adam Hilger, NY ,**1989**.
[13] G. J. Martyna, M. L. Klein, M. Tuckerman, *The Journal of Chemical Physics* **1992**, *97*, 2635.
[14] Y. Liang, F. Zhao, Z. Cheng, Y. Deng, Y. Xiao, H. Cheng, P. Zhang, Y. Huang, H. Shao, L. Qu, *Energy Environmental. Science* **2018**, *11*, 1730.
[15] D. Shen, M. Xiao, G. Zou, L. Liu, W. W. Duley, Y. N. Zhou, *Advanced Materials* **2018**, *30*, 1705925.
[16] Y. Huang, H. Cheng, C. Yang, P. Zhang, Q. Liao, H. Yao, G. Shi, L. Qu, *Nature Communications* **2018**, *9*, 1.
[17] N. Chen, Q. Liu, C. Liu, G. Zhang, J. Jing, C. Shao, Y. Han, L. Qu, *Nano Energy* **2019**, *65*, 104047.
[18] Y. Huang, H. Cheng, C. Yang, H. Yao, C. Li, L. Qu, *Energy Environmental. Science* **2019**, *12*, 1848.
[19] W. Yang, X. Li, X. Han, W. Zhang, Z. Wang, X. Ma, M. Li, C. Li, *Nano Energy* **2020**, *71*, 104610.
[20] X. Liu, H. Gao, J. E. Ward, X. Liu, B. Yin, T. Fu, J. Chen, D. R. Lovley, J. Yao, *Nature* **2020**, *578*, 550.
[21] G. Ren, Z. Wang, B. Zhang, X. Liu, J. Ye, Q. Hu, S. Zhou, *Nano Energy* **2021**, *89*, 106361.
[22] D. He, Y. Yang, Y. Zhou, J. Wan, H. Wang, X. Fan, Q. Li, H. Huang, *Nano Energy* **2021**, *81*, 105630.
[23] H. Wang, Y. Sun, T. He, Y. Huang, H. Cheng, C. Li, D. Xie, P. Yang, Y. Zhang, L. Qu, *Nature. Nanotechnology.* **2021**, *16*, 811.
[24] Q. Zhao, Y. Jiang, Z. Duan, Z. Yuan, J. Zha, Z. Wu, Q. Huang, Z. Zhou, H. Li, F. He, Y. Su, C. Tan, H. Tai, *Chemical Engineering Journal* **2022**, *438*, 135588.
[25] H. Wang, T. He, X. Hao, Y. Huang, H. Yao, F. Liu, H. Cheng, L. Qu, *Nature Communications* **2022**, *13*, 1.
[26] T. He, H. Wang, B. Lu, T. Guang, C. Yang, Y. Huang, H. Cheng, L. Qu, *Joule* **2023**, *7*, 935.
[27] G. Ma, W. Li, X. Zhou, X. Wang, M. Cao, W. Ma, J. Wang, H. Yu, S. Li, Y. Chen, *ACS Applied Polymer Materials* **2024**, *12*, 7066.
[28] F. Gong, J. Song, H. Chen, H. Li, R. Huang, Y. Jing, P. Yang, J. Feng, R. Xiao, *Front. Energy* **2024**, *18*.
